# A global analysis of Y-chromosomal haplotype diversity for 23 STR loci

**DOI:** 10.1016/j.fsigen.2014.04.008

**Published:** 2014-09

**Authors:** Josephine Purps, Sabine Siegert, Sascha Willuweit, Marion Nagy, Cíntia Alves, Renato Salazar, Sheila M.T. Angustia, Lorna H. Santos, Katja Anslinger, Birgit Bayer, Qasim Ayub, Wei Wei, Yali Xue, Chris Tyler-Smith, Miriam Baeta Bafalluy, Begoña Martínez-Jarreta, Balazs Egyed, Beate Balitzki, Sibylle Tschumi, David Ballard, Denise Syndercombe Court, Xinia Barrantes, Gerhard Bäßler, Tina Wiest, Burkhard Berger, Harald Niederstätter, Walther Parson, Carey Davis, Bruce Budowle, Helen Burri, Urs Borer, Christoph Koller, Elizeu F. Carvalho, Patricia M. Domingues, Wafaa Takash Chamoun, Michael D. Coble, Carolyn R. Hill, Daniel Corach, Mariela Caputo, Maria E. D’Amato, Sean Davison, Ronny Decorte, Maarten H.D. Larmuseau, Claudio Ottoni, Olga Rickards, Di Lu, Chengtao Jiang, Tadeusz Dobosz, Anna Jonkisz, William E. Frank, Ivana Furac, Christian Gehrig, Vincent Castella, Branka Grskovic, Cordula Haas, Jana Wobst, Gavrilo Hadzic, Katja Drobnic, Katsuya Honda, Yiping Hou, Di Zhou, Yan Li, Shengping Hu, Shenglan Chen, Uta-Dorothee Immel, Rüdiger Lessig, Zlatko Jakovski, Tanja Ilievska, Anja E. Klann, Cristina Cano García, Peter de Knijff, Thirsa Kraaijenbrink, Aikaterini Kondili, Penelope Miniati, Maria Vouropoulou, Lejla Kovacevic, Damir Marjanovic, Iris Lindner, Issam Mansour, Mouayyad Al-Azem, Ansar El Andari, Miguel Marino, Sandra Furfuro, Laura Locarno, Pablo Martín, Gracia M. Luque, Antonio Alonso, Luís Souto Miranda, Helena Moreira, Natsuko Mizuno, Yasuki Iwashima, Rodrigo S. Moura Neto, Tatiana L.S. Nogueira, Rosane Silva, Marina Nastainczyk-Wulf, Jeanett Edelmann, Michael Kohl, Shengjie Nie, Xianping Wang, Baowen Cheng, Carolina Núñez, Marian Martínez de Pancorbo, Jill K. Olofsson, Niels Morling, Valerio Onofri, Adriano Tagliabracci, Horolma Pamjav, Antonia Volgyi, Gusztav Barany, Ryszard Pawlowski, Agnieszka Maciejewska, Susi Pelotti, Witold Pepinski, Monica Abreu-Glowacka, Christopher Phillips, Jorge Cárdenas, Danel Rey-Gonzalez, Antonio Salas, Francesca Brisighelli, Cristian Capelli, Ulises Toscanini, Andrea Piccinini, Marilidia Piglionica, Stefania L. Baldassarra, Rafal Ploski, Magdalena Konarzewska, Emila Jastrzebska, Carlo Robino, Antti Sajantila, Jukka U. Palo, Evelyn Guevara, Jazelyn Salvador, Maria Corazon De Ungria, Jae Joseph Russell Rodriguez, Ulrike Schmidt, Nicola Schlauderer, Pekka Saukko, Peter M. Schneider, Miriam Sirker, Kyoung-Jin Shin, Yu Na Oh, Iulia Skitsa, Alexandra Ampati, Tobi-Gail Smith, Lina Solis de Calvit, Vlastimil Stenzl, Thomas Capal, Andreas Tillmar, Helena Nilsson, Stefania Turrina, Domenico De Leo, Andrea Verzeletti, Venusia Cortellini, Jon H. Wetton, Gareth M. Gwynne, Mark A. Jobling, Martin R. Whittle, Denilce R. Sumita, Paulina Wolańska-Nowak, Rita Y.Y. Yong, Michael Krawczak, Michael Nothnagel, Lutz Roewer

**Affiliations:** a Department of Forensic Genetics, Institute of Legal Medicine and Forensic Sciences, Charité-Universitätsmedizin, Berlin, Germany; b Department of Statistical Genetics and Bioinformatics, Cologne Center for Genomics, University of Cologne, Germany; c Institute of Molecular Pathology and Immunology of the University of Porto (IPATIMUP), Porto, Portugal; d Department of Biology, Faculty of Sciences, University of Porto, Portugal; e Philippine National Police Crime Laboratory, Quezon City, Philippines; f Institut für Rechtsmedizin, Ludwig-Maximilians-Universität, München, Germany; g The Wellcome Trust Sanger Institute, Wellcome Trust Genome Campus, Hinxton, UK; h Department of Forensic Medicine, University of Zaragoza, Spain; i GenoID Forensic DNA Laboratory, Department of Genetics, Eötvös Loránd University, Budapest, Hungary; j Institut für Rechtsmedizin, Universität Basel, Switzerland; k Department of Forensic and Analytical Science, King's College London, London, UK; l Forensic Sciences Department, Poder Judicial, Heredia, Costa Rica; m Landeskriminalamt Baden-Württemberg, Germany; n Institute of Legal Medicine, Innsbruck Medical University, Innsbruck, Austria; o Penn State Eberly College of Science, University Park, PA, USA; p Institute of Applied Genetics and Department of Molecular and Medical Genetics, Ft. Worth, USA; q Center of Excellence in Genomic Medicine Research (CEGMR), King Abdulaziz University, Jeddah, Saudi Arabia; r Forensische Genetik, Kantonsspital Aarau AG, Switzerland; s Laboratorio de Diagnósticos por DNA, Instituto de Biologia, Universidade do Estado do Rio de Janeiro, Brazil; t Human DNA Diagnostics Laboratory, Beirut, Lebanon; u National Institute of Standards and Technology, Gaithersburg, USA; v Universidad de Buenos Aires, Facultad de Farmacia y Bioquimica, Servicio de Huellas Digitales Genetica and CONICET (National Scientific and Technical Research Council), Buenos Aires, Argentina; w University of the Western Cape, Biotechnology Department, Forensic DNA Laboratory, Cape Town, South Africa; x KU Leuven, Department of Imaging & Pathology, Laboratory of Forensic Genetics and Molecular Archaeology, Leuven, Belgium; y Centre of Molecular Antropology For Ancient DNA Studies, Department of Biology, University of Rome Tor Vergata, Italy; z Collaborative Innovation Center of Judicial Civilization, Institute of Evidence Law and Forensic Science, China University of Political Science and Law, Beijing, China; aa Institute of Forensic Medicine, Medical University, Wroclaw, Poland; ab Illinois State Police, Research & Development Laboratory, Springfield, USA; ac Department of Forensic Medicine and Criminology, University of Zagreb, Croatia; ad University Center of Legal Medicine, Lausanne-Geneva, Lausanne, Switzerland; ae Forensic Science Centre “Ivan Vucetic”, General Police Directorate, Ministry of Interior, Zagreb, Croatia; af Institut für Rechtsmedizin, Universität Zürich, Switzerland; ag National Forensic Laboratory, Ljubljana, Slovenia; ah Department of Legal Medicine, Faculty of Medicine, University of Tsukuba, Japan; ai Institute of Forensic Medicine, West China School of Basic Science and Forensic Medicine Sichuan University, Chengdu, China; aj Molecular Biology and Forensic Genetics Laboratory, Shantou University Medical College, Shantou, China; ak Institut für Rechtsmedizin, Universität Halle, Germany; al Institute for Forensic Medicine and Criminalistics, Medical Faculty, University “Ss. Cyril and Methodius“, Skopje, Macedonia; am Institut für Rechtsmedizin, Universitätsmedizin Greifswald, Germany; an Forensic Laboratory for DNA Research, Department of Human Genetics, Leiden University Medical Center, Leiden, The Netherlands; ao Subdivision of Biological and Biochemical Examinations and Analyses F.S.D. – Hellenic Police, Athens, Greece; ap Institute for Genetic Engineering and Biotechnology, Sarajevo, Bosnia and Herzegovina; aq Institut für Rechtsmedizin, Universität Rostock, Germany; ar Molecular Biology Laboratory, American University of Science and Technology Beirut, Lebanon and School of Criminal Justice, University of Lausanne, Switzerland; as Laboratorio de Análisis de ADN, FCM - National University of Cuyo, Mendoza, Argentina; at Instituto Nacional de Toxicología y Ciencias Forenses, Madrid, Spain; au Departamento de Biologia, Universidade de Aveiro, Portugal; av National Research Institute of Police Science, Chiba, Japan; aw Instituto de Biologia, Universidade Federal do Rio de Janeiro and DIMAV/INMETRO, Brazil; ax Instituto de Biologia do Exército, Rio de Janeiro, Brazil; ay Instituto de Biofísica Carlos Chagas Filho, Universidade Federal do Rio de Janeiro, Brazil; az Institut für Rechtsmedizin, Universität Leipzig, Germany; ba School of Forensic Medicine, Kunming Medical University, Kunming, China; bb Department of Criminal Investigation, Xuanwei Public Security Bureau, Xuanwei, China; bc Department of Criminal Investigation, Yunnan Provincial Public Security Bureau, Kunming, China; bd BIOMICs Research Group, Universidad del País Vasco, Vitoria, Spain; be Section of Forensic Genetics, Department of Forensic Medicine, Faculty of Health and Medical Sciences, University of Copenhagen, Denmark; bf Section of Legal Medicine, Università Politecnica delle Marche, Ancona, Italy; bg DNA Laboratory, Institute for Forensic Medicine, Network of Forensic Science Institutes, Ministry of Public Administration and Justice, Budapest, Hungary; bh Forensic Genetics Laboratory, Institute of Forensic Medicine, Medical University of Gdansk, Poland; bi Department of Medical and Surgical Sciences (DIMEC), Institute of Legal Medicine, School of Medicine, University of Bologna, Italy; bj Department of Forensic Medicine, Medical University of Bialystok, Poland; bk Department of Forensic Medicine, Medical University Poznan, Poland; bl Unidade de Xenética Forense, Instituto de Ciencias Forenses, Grupo de Medicina Xenómica, Facultade de Medicina, Universidade de Santiago de Compostela, Spain; bm Forensic Genetics Laboratory, Institute of Legal Medicine, Università Cattolica del Sacro Cuore, Rome, Italy; bn Department of Zoology, University of Oxford, Oxford, UK; bo PRICAI-Fundación Favaloro, Buenos Aires, Argentina; bp Forensic Genetics Laboratory, Department of Human Morphology and Biomedical Sciences, Università degli Studi di Milano, Italy; bq Interdisciplinary Department of Medicine, Section of Legal Medicine, University of Bari, Italy; br Department of Medical Genetics, Warsaw Medical University, Poland; bs Department of Forensic Medicine, Warsaw Medical University, Poland; bt Department of Public Health Sciences and Pediatrics, University of Turin, Italy; bu Department of Forensic Medicine, University of Helsinki, Finland; bv DNA Analysis Laboratory, Natural Sciences Research Institute, University of the Philippines Diliman, Philippines; bw Institute of Biological Sciences, University of the Philippines Los Baños, Laguna, Philippines; bx Institut für Rechtsmedizin, Universitätsklinikum Freiburg, Germany; by Department of Forensic Medicine, University of Turku, Finland; bz Institute of Legal Medicine, Faculty of Medicine, University of Cologne, Germany; ca Department of Forensic Medicine, Yonsei University College of Medicine, Seoul, South Korea; cb Athens Dept. of Legal Medicine, DNA Analysis Laboratory, Athens, Greece; cc Department of Basic Medical Sciences, University of the West Indies, Kingston, Jamaica; cd Laboratorio Genetix S.A., Panamá, Panama; ce Laboratory of Forensic Genetics, Institute of Criminalistics, Prague, Czech Republic; cf Department of Forensic Genetics and Forensic Toxicology, National Board of Forensic Medicine, Linköping, Sweden; cg Sezione di Medicina Legale, Dipartimento di Medicina e Sanità Pubblica, Università degli Studi di Verona, Italy; ch Istituto di Medicina Legale, Universitá degli Studi di Brescia, Italy; ci Department of Genetics, University of Leicester, UK; cj Genomic Engenharia Molecular Ltda., Sao Paulo, Brazil; ck Institute of Forensic Research, Krakow, Poland; cl Defence Medical & Environmental Research Institute, DSO National Laboratories, Singapore; cm Institute of Medical Informatics and Statistics, Christian-Albrechts University Kiel, Germany

**Keywords:** Gene diversity, Discriminatory power, AMOVA, Population structure, Database

## Abstract

In a worldwide collaborative effort, 19,630 Y-chromosomes were sampled from 129 different populations in 51 countries. These chromosomes were typed for 23 short-tandem repeat (STR) loci (DYS19, DYS389I, DYS389II, DYS390, DYS391, DYS392, DYS393, DYS385ab, DYS437, DYS438, DYS439, DYS448, DYS456, DYS458, DYS635, GATAH4, DYS481, DYS533, DYS549, DYS570, DYS576, and DYS643) and using the PowerPlex Y23 System (PPY23, Promega Corporation, Madison, WI). Locus-specific allelic spectra of these markers were determined and a consistently high level of allelic diversity was observed. A considerable number of null, duplicate and off-ladder alleles were revealed. Standard single-locus and haplotype-based parameters were calculated and compared between subsets of Y-STR markers established for forensic casework. The PPY23 marker set provides substantially stronger discriminatory power than other available kits but at the same time reveals the same general patterns of population structure as other marker sets. A strong correlation was observed between the number of Y-STRs included in a marker set and some of the forensic parameters under study. Interestingly a weak but consistent trend toward smaller genetic distances resulting from larger numbers of markers became apparent.

## 1 Introduction

Early Y-chromosomal short-tandem repeat (STR) markers used in forensic practice either were discovered in cloning experiments [1,2] or were retrieved in silico from the Genome Database (GDB) [3]. These markers include, for example, the nine loci constituting the ‘minimal haplotype’ (MHT) marker set [4], which still forms the core of all Y-STR kits in current forensic use but at the same time represents a rather heterogeneous and somewhat random choice of markers with different population genetic properties. Meanwhile, the complete euchromatic region of the human Y-chromosome has been sequenced [5] and, with the human reference sequence at hand [6], a more systematic search for potentially useful Y-STRs became feasible. Thus, a recent study by Ballantyne et al. [7] identified 167 novel Y-STRs and combined those 13 with the highest mutation rate in a set of so-called “rapidly mutating” (RM) markers. The same study also revealed that between 50% and 100% of pairs of related men (at most 20 meioses apart) can be resolved by at least one mutation of these RM Y-STRs. Such results indicated that low level haplotype sharing between patrilineal relatives pertain to combinations of RM Y-STRs in general, thereby overcoming a limitation of using Y-STR typing of forensic evidence. However, the multi-copy structure of some of the most mutable Y-STRs renders genotyping difficult and often unreliable so that the RM approach has not yet become fully integrated into forensic casework.

The PowerPlex^®^Y23 System (PPY23, Promega Corporation, Madison, WI) is a five-dye Y-STR multiplex designed for genotyping male samples at 23 loci. It is intended to be used in forensic casework, kinship analysis and population genetic studies. Advantageous features such as short fragment length and an uninterrupted repeat structure were taken into account when constructing the kit. Six new markers (DYS481, DYS533, DYS549, DYS570, DYS576 and DYS643), two of which (DYS570 and DYS576) categorized as “rapidly mutating” [7], were added to an existing panel of 17 markers, already contained within the Yfiler^®^kit (Yfiler, Life Technologies, Foster City, CA). The first studies employing the new PPY23 kit revealed a markedly increased haplotype diversity and discriminatory power in comparison to other marker sets, including the MHT, SWGDAM (recommended by the US-based Scientific Working Group for DNA Analysis Methods, www.swgdam.org), PowerPlex^®^Y12 (PPY12) and Yfiler panels [8–10]. Here is presented a much more comprehensive analysis of almost 20,000 Y-chromosomes, sampled from 129 populations in 51 countries worldwide and genotyped between September 2012 and June 2013. The gain in information for forensic casework was assessed from that provided by the PPY23 panel and compared to the Yfiler, PPY12, SWGDAM and MHT panels. Possible population differences [11] were determined based on genetic distances between single populations as well as between continental groups. All haplotype data used in the study are publicly available at the Y Chromosome Haplotype Reference Database (YHRD) website (www.yhrd.org).

## 2 Materials and methods

### 2.1 DNA sample collection

**Fig. 1:**
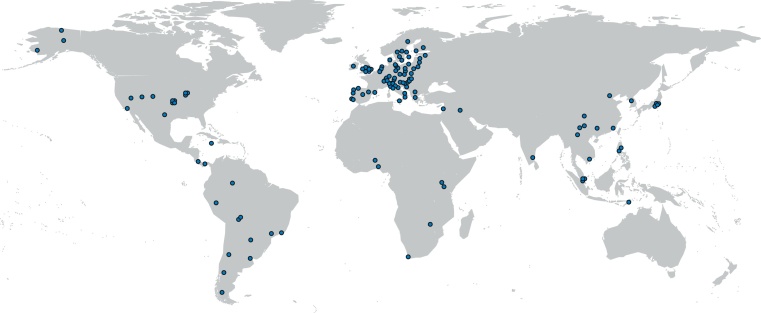
Geographic distribution of sampling sites. Male samples (*n* = 19,630) were collected at 129 sites on five continents.

Between 9/2012 and 6/2013, a total of 19,630 Y-STR haplotypes were compiled in 84 participating laboratories. In particular, unrelated males were typed from 129 populations in 51 countries worldwide (Fig. 1; Table S1 and Fig. S1). Most of the samples had been typed before for smaller marker sets, mostly the Yfiler panel (DYS19, DYS389I, DYS389II, DYS390, DYS391, DYS392, DYS393, DYS385ab, DYS437, DYS438, DYS439, DYS448, DYS456, DYS458, DYS635 and GATAH4) and the corresponding haplotypes had been deposited in YHRD. All samples were now also typed for the full PPY23 panel (17 markers in Yfiler plus the loci DYS481, DYS533, DYS549, DYS570, DYS576 and DYS643), and samples from 40 populations were typed completely anew. The YHRD accession numbers of the 51 populations are given in Supplementary Table S2. DNA samples were genotyped following the manufacturer's instructions [12] with the occasional adaptation to prevailing laboratory practice. Populations were placed into five groups (‘meta-populations’) according to either (i) continental residency (445 African, 3458 Asian, 11,968 European, 1183 Latin American, 2576 North American) or (ii) continental ancestry, defined as the historical continental origin of the source population (1294 African, 3976 Asian, 12,585 European, 558 Native American, 1217 Mixed American) (Table S2).

### 2.2 Quality control

Each participating laboratory passed a quality assurance test that is compulsory for all Y-STR studies to be publicized by, and uploaded to, YHRD. In particular, each laboratory analyzed five anonymized samples of 10 ng DNA each, using the PowerPlex^®^Y23 kit. The resulting profiles were evaluated centrally by the Department of Forensic Genetics at the Charité – Universitätsmedizin Berlin, Germany. All haplotypes previously uploaded to YHRD were automatically aligned to the corresponding PPY23 profiles and assessed for concordance. Plausibility checks, including the allelic range and the occurrence of intermediate alleles, were performed for the six novel loci (i.e. DYS481, DYS533, DYS549, DYS570, DYS576 and DYS643).

### 2.3 Forensic parameters

Forensic parameters were calculated for all samples (*n* = 19,630) and for all 23 markers of the PPY23 kit. To this end, DYS389II alleles were encoded by the difference, henceforth labeled DYS389II.I, between the total repeat number at DYS389II and the repeat number at DYS389I. DYS385ab haplotypes were treated as single alleles thereby ignoring the internal order of its two component alleles. Forensic parameters were calculated for the study as a whole and for meta-populations defined according to the continental or ethnic origin of the samples (see above).

In particular, allele frequencies and haplotype frequencies were estimated using the counting method. Single-marker genetic diversity (GD) was calculated as $\text{GD}=n\left(1-\sum p_{i}^{2}\right)/(n-1)$, following Nei [13,14], where *n* and *p*_*i*_ denote the total number of samples and the relative frequency of the *i*-th allele, respectively. Haplotype diversity (HD) was calculated analogous to GD. Match probability (MP) was calculated as the sum of squared haplotype frequencies. The discrimination capacity (DC) was defined as the ratio between the number of different haplotypes and the total number of haplotypes. To benchmark the practical utility of the PPY23 panel for forensic casework, all haplotype-based analyses were repeated for various subsets of Y-STRs, namely the MHT (9 loci), SWGDAM (11 loci), PPY12 (12 loci) and Yfiler marker panels (17 loci). The Yfiler and PPY23 panels also were compared to one another after confining both panels to Y-STRs with an amplicon length <220 bp.

### 2.4 Population differences

The extent of population genetic structure in our data was assessed by means of analysis of molecular variance (AMOVA). More specifically, genetic distances between groups of males were quantified by *R*_ST_, thereby taking the evolutionary distance between individual Y-STR haplotypes into account [15,16]. The DYS385ab marker was not included in the AMOVA because it does not allow easy calculation of evolutionary distances. Samples carrying a deletion, a null allele, an intermediate allele (i.e. an incomplete repeat unit), a duplication or a triplication at one or more markers were excluded from the AMOVA (*n* = 705, 3.6%), leaving 18,925 haplotypes for analysis (Supplementary Table S2). *R*_ST_ values resulting from continental grouping were compared among the PPY23, Yfiler, PPY12, SWGDAM, and MHT panels. Multidimensional scaling (MDS) analysis served to visualize differences in Y-STR genetic variation between populations and was based upon pairwise linearized *R*_ST_ values for PPY23, that is *R*_ST_/(1 − *R*_ST_). MDS is commonly used to investigate genetic similarities between populations and has been described in detail elsewhere [17]. First, MDS analyses were performed for one to 10 dimensions considering either all 129 populations or the 68 European populations alone. For each population set and each dimensionality, a stable MDS solution was obtained iteratively reducing Kruskal's stress value [17,18] until it remained nearly unchanged (i.e. until the ratio of consecutive stress values exceeded 0.99). The optimal dimensionality then was determined for each population set by a visual ‘scree’ test.

All analyses were performed using R statistical software v2.15.3 [19] or Arlequin v3.5.1.2 [20], as appropriate. In particular, Arlequin was employed to estimate *R*_ST_ values and for randomization-based significance testing of genetic distances (10,000 replicates per comparison) [20]. Covariance components (i.e. percentages of variation) associated with different levels of geographic grouping were tested for statistical significance using a non-parametric permutation approach described by Excoffier et al. [15] (10,000 replicates). For MDS, R package *vegan* v.2.0-10 was used [21]. Geographic maps were generated in R using packages *maps* v.2.3-6 [22] and *mapdata* v.2.2-2 [23]. The latter is based upon an amended version of the CIA World Data Bank II. In order to perform spatial interpolation, we estimated the spatial model using random Gaussian fields, while conventional kriging was used for interpolation, as implemented in the likfit and krige.conv functions from the geoR v1.7-4 [24,25].

## 3 Results

### 3.1 Single-locus analysis

**Fig. 2:**
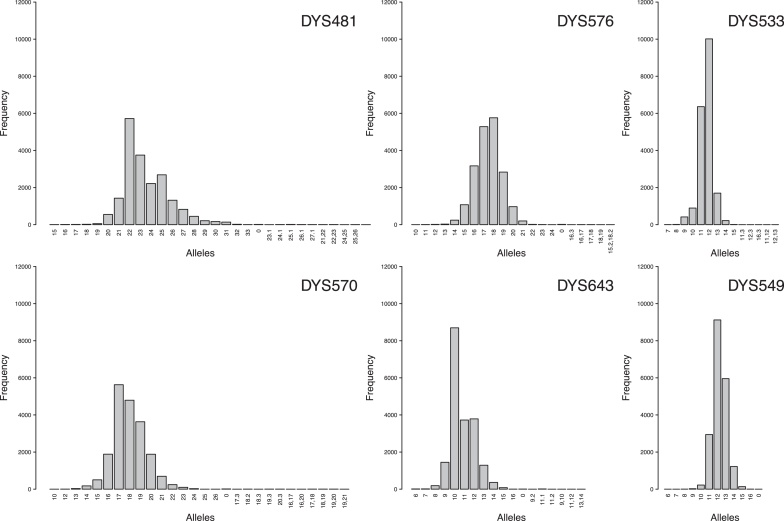
Allele distribution of PPY23-specific loci.

**Fig. 3:**
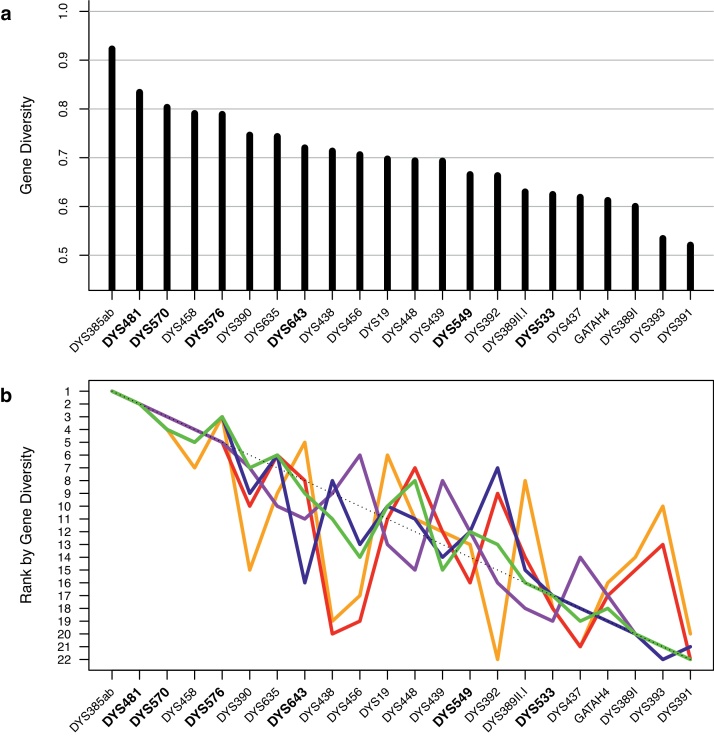
Ranking of PPY23 markers by gene diversity (GD). For the calculation of GD, DYS385ab was treated as a single marker. DYS389II.I equals the difference between DYS389II and DYS389I. PPY23-specific markers are given in bold. (a) Rank within the whole data set; (b) rank within continental residency groups, i.e. Africa (orange; *n* = 445), Asia (red; *n* = 3458), Europe (magenta; *n* = 11,968), Latin America (blue; *n* = 1183) or North America (green; *n* = 2576). (For interpretation of the references to color in this figure legend, the reader is referred to the web version of this article.)

A high level of genetic diversity was observed in our study at all 23 Y-STRs of the PPY23 panel. Some 521 different alleles were observed in the 19,630 Y-chromosomes analyzed, with a median number of 16 alleles per marker and a range of 10 (DYS391) to 31 (DYS458; Table S3). Marker DYS385ab showed 146 different allele combinations (i.e. unordered haplotypes). A total of 133 null alleles occurred at 17 of the 23 loci, 75 intermediate alleles (18 loci) and 69 copy-number variants (21 loci; 57 duplications excluding all duplicates at DYS385ab, 11 triplications, one quadruplication). Of the six markers that distinguish PPY23 from Yfiler, the DYS481 and DYS570 markers showed the largest numbers of different alleles (30 and 28, respectively; Fig. 2). Gene diversity (GD) values exceeded 0.5 for all 23 markers, 0.6 for 21 (91.3%) and even 0.7 for 10 (43.5%) markers (Fig. 3a; Table S4). While of the 17 markers in common with the Yfiler kit, markers DYS385ab (GD = 0.923) on the one hand, and DYS391 (0.521) and DYS393 (0.534) on the other marked the extremes of the GD distribution, four of the six PPY23-specific markers, namely DYS481, DYS570, DYS576 and DYS643, ranked near the top, with GD values exceeding 0.72. Notably, some loci ranked differently with respect to GD in different continental (Fig. 3b) or ancestry groups (Fig. S2), most prominently with regard to the African meta-population (Table S4). For example, the DYS390, DYS438 and DYS392 markers were found to be less variable in Africa than, for example, in Europe. Of the six PPY23-specific markers, all but DYS643 showed similar GD values on most continents. The DYS643 marker was found to be more variable in Africans, but less variable in Native Americans from Latin America, than in the other continental groups (Fig. S2).

### 3.2 Variant and off-ladder alleles

**Fig. 4:**
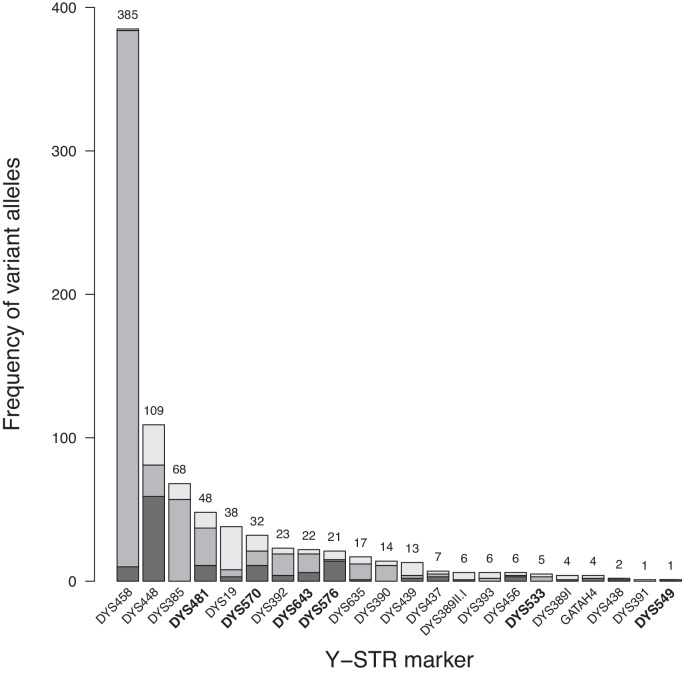
Marker-wise frequency of variant alleles. PPY23-specific markers are given in bold. Light gray: copy number variants; gray: intermediate alleles; dark gray: null allele.

Variant alleles not representing simple repetitions of the respective STR motif occurred at all loci (Table S3). This included null alleles, likely due to a deletion or primer site mutation, intermediate alleles comprising fractional repeats, and copy-number variants such as duplications and triplications of the whole locus. All variant alleles were confirmed by retyping or sequencing at the laboratory that had performed the original STR typing. The proportion of variant alleles differed greatly among markers (Fig. 4), with DYS458 showing the highest (*n* = 385) and DYS391 and DYS549 showing the lowest number (*n* = 1). Four of the six PPY23-specific markers (DYS481, DYS570, DYS576 and DYS643) had comparatively high numbers of variant alleles. Only two single non-fractional off-ladder alleles (allele 6 at GATAH4, allele 15 at DYS481) were observed in this study. On the other hand, only five of the 19 intermediate alleles observed for the six PPY23-specific markers (18.2, 18.3, 19.3 and 20.3 at DYS570, 11.1 at DYS643) were included in the bin set of the allelic ladder (Table S3).

Some 75 different intermediate alleles occurred at one of 18 Y-STR loci and were seen in 550 samples (Table S3). DYS458 was the locus with the highest proportion of intermediate alleles (16 different in 374 samples), followed by DYS385ab (12 different in 57 samples) and DYS448 (8 different in 23 samples). Of the PPY23-specific markers, DYS481 had the highest number of different intermediate alleles (5 in 26 samples) of which 25.1 was the most frequent (*n* = 13). The structure of 11.1 at the DYS643 marker (observed in 11 samples in our study) has been reported previously [26] and is included already in the PPY23 allelic ladder.

A total of 133 null alleles were observed at 17 loci (Table S3), which corresponds to an overall frequency of 0.03%. The DYS448 locus showed the highest number of null alleles (*n* = 59), followed by PPY23-specific markers DYS576 (*n* = 14), DYS481 (*n* = 11) and DYS570 (*n* = 11). In nine samples, a large deletion was detected at Yp11.2 encompassing the *AMELY* region that removed four adjacent loci (DYS570, DYS576, DYS458 and DYS481). All these samples were of Asian ancestry, namely Indians from Singapore, Tamils from Southern India and British Asians with reported origins from Pakistan or India, where this type of deletion is frequent [27,28]. Furthermore, two of the nine samples also carried a null allele at DYS448 [29]. Upon retyping with autosomal kits, all these samples showed a deletion of the *AMELY* gene locus. Another large deletion located at Yq11 and encompassing the *AZFa* region [30] affected two adjacent loci (DYS389I/II and DYS439) and was detected in one African American sample. Concomitant null alleles at three loci were observed in a Han Chinese sample (DYS448, DYS458, GATAH4) and an Indian sample (DYS392, DYS448, DYS549). The DYS448 and DYS456 markers were both not amplifiable in an Iraqi sample. Furthermore, null alleles were observed at DYS576 in four samples of Asian ancestry from the UK.

There were 69 copy number variants, mostly duplications, observed at 21 loci (all except DYS438 and DYS549). Copy number variants were most abundant at the markers DYS19 (*n* = 30) and DYS448 (28), followed by DYS481 and DYS570 (11 each; Table S3). Note that, at DYS385ab, only copy numbers larger than two are conventionally counted. One triplication each of the DYS19 and DYS448 markers was observed in African American samples and a duplication comprising two intermediate alleles (15.2 and 18.2) at the DYS576 marker occurred in a European American sample. Duplications of several consecutive loci in the *AZFa* region [31] were detected in three samples at DYS389I/II and DYS439 in two samples and additionally including DYS437 in a Hispanic American sample. A previously published duplication affecting the DYS570 and DYS576 markers [10] was found a second time in a German sample from our study.

### 3.3 Haplotype analysis

The 23 markers of the PPY23 panel were evaluated with respect to their haplotype diversity (HD), discrimination capacity (DC) and other forensic parameters such as random match probability (MP). In total, 18,860 different haplotypes were observed (Table 1). Of the 19,630 samples analyzed, 18,237 (92.9%) carried a unique haplotype. The most frequent haplotype was detected 11 times across three different populations, namely the Athapaskans, Estonians and Finns. Finland, Alaska and Kenya had the highest numbers of haplotypes occurring more than once (Table 1). Notably, eight Maasai individuals from Kinyawa (Kenya) and seven Xhosa from South Africa shared an identical haplotype, respectively. Haplotypes that were observed at least four times in a population were found in Reutte (Austria, Tyrolean; *n* = 1), Finland (Finnish; *n* = 5), Netherlands (Dutch; *n* = 1), Xuanwei (China, Han; *n* = 2), Kinyawa (Kenya, Maasai; *n* = 5), South Africa (Xhosa; *n* = 2), Peru (Peruvian; *n* = 1), Northern Alaska (USA, Inupiat; *n* = 5) and Western Alaska (USA, Yupik; *n* = 1) (data not shown).

Of the meta-populations formed according to continental residency, Asia showed the highest DC (>0.97), followed by Europe and Latin America (DC ∼ 0.96), and finally Africa (DC ∼ 0.85; Table S5). Grouping by continental ancestry yielded similar DC values of >0.96 for Asians, Europeans and Mixed Americans. However, a decrease in DC was observed for Native Americans (0.83) and an increase for samples of African ancestry (0.94; Table S5). Notably, 42 out of the 129 population samples (32.6%) contained only unique PPY23 haplotypes (‘complete resolution’), namely seven Asian, 23 European, six Latin America and six North America (i.e. no African populations).

### 3.4 Comparative analysis of five forensic Y-STR marker sets

We compared the haplotype-based forensic parameters for five different sets of Y-STR markers commonly used in forensic practice, namely MHT, SWGDAM, PPY12, Yfiler and PPY23. Not surprisingly, a strictly monotonous relationship emerged among all forensic parameters and the number of markers included in a panel (Table 2). Both the number of different and unique haplotypes increased almost linearly with marker number in the overall sample (Pearson's correlation coefficient *r* = 0.97 and *r* = 0.98, respectively). On average, each additional marker generated 754 new different haplotypes (*p* = 0.005 from linear regression) and 888 new unique haplotypes (*p* = 0.003) in the overall sample. The proportion of unique haplotypes worldwide increased from 31.0% for MHT via 77.8% for Yfiler to 92.9% for PPY23 (Table 2). Correspondingly, DC increased from 43.0% for MHT to 96.1% for PPY23 (*r* = 0.97). HD showed a similar trend (*r* = 0.81) whereas MP decreased rapidly with increasing marker number (*r* = −0.81). Similar trends were observed in the meta-populations defined according to both continental origin and ancestry (Table S5). In summary, an increasing number of markers was found to be associated with an almost linear increase of all forensic parameters used to discriminate among individuals.

### 3.5 Comparison of short amplicon subsets of Yfiler and PPY23

The forensic parameters were compared of Y-STRs that have amplicons shorter than 220 bp and that are included in Yfiler (DYS456, DYS389I, DYS458, DYS19, DYS393, DYS391, GATAH4, and DYS437) or PPY23 (DYS576, DYS389I, DYS391, DYS481, DYS570, DYS635, DYS393, and DYS458). A substantially stronger discriminatory power of PPY23 compared to Yfiler was evident for these short haplotypes, mostly due to the higher diversity of PPY23-specific markers DYS576, DYS481, DYS570 and DYS635. In particular, DC and the number of different short haplotypes were nearly twice as high for PPY23 as for Yfiler whereas MP was more than 4-fold smaller (Table 3).

### 3.6 Population structure

**Fig. 5:**
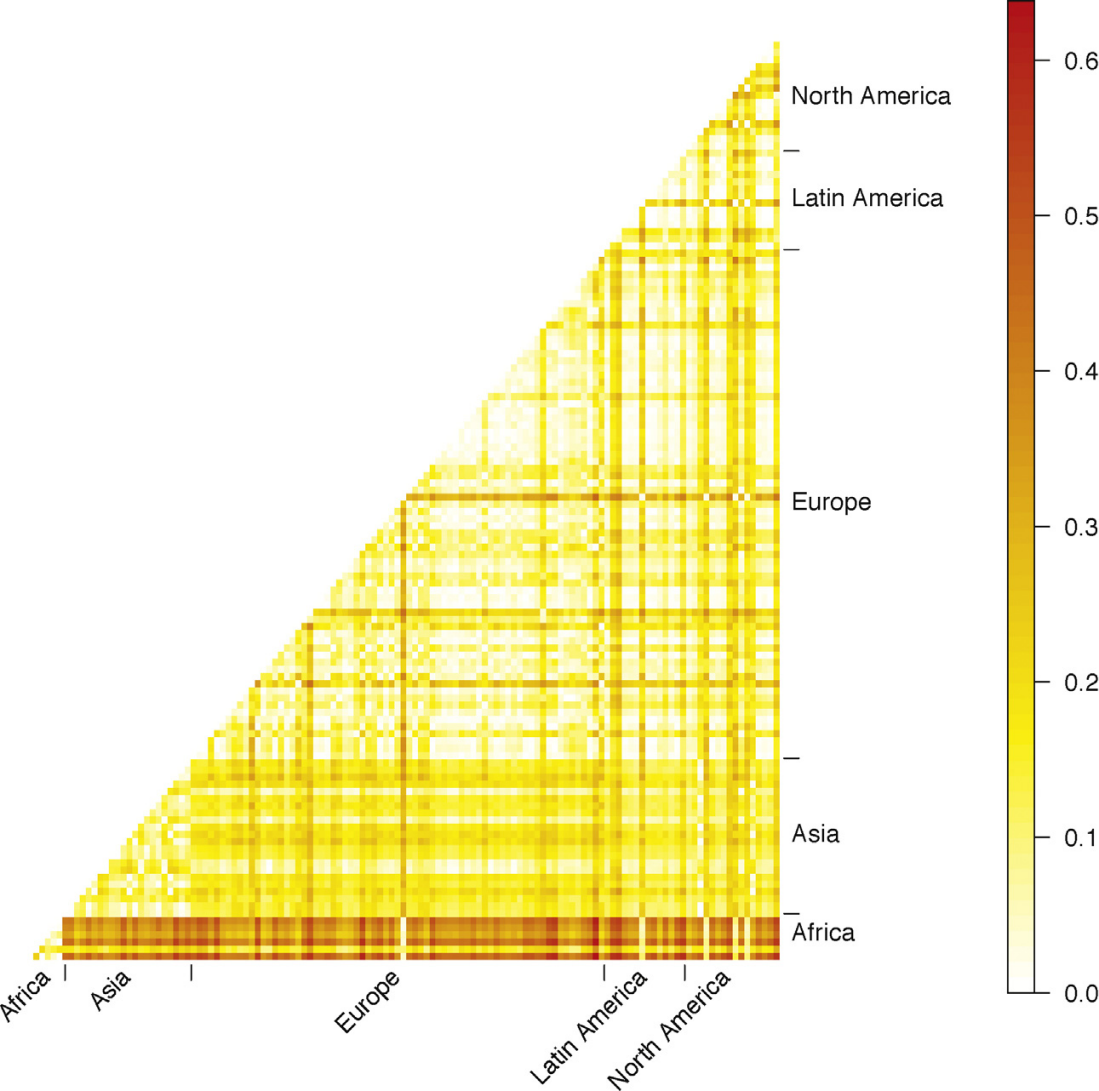
Genetic distances between populations. Pairwise *R*_ST_ values for PPY23 were calculated between all 129 populations and grouped by continental residency.

At the continental level, by far the largest genetic distances were observed between the African meta-population and the other four groups (all *R*_ST_ > 0.2 for PPY23, *p* < 10^−4^). Genetic distances between non-African meta-populations were much smaller although still significant (*p* < 10^−4^). The smallest genetic distance was noted for North and Latin America (*R*_ST_ = 0.009 with PPY23; Table 4). Similarly, at the population level, pairs of African and non-African populations showed much larger genetic distances (with *R*_ST_ > 0.3 in some instances) than pairs of non-African populations or African populations (Fig. 5, Table S6). Upon AMOVA, 85.1% of the overall PPY23 haplotype variation was within populations, 9.1% was among populations within meta-populations, defined according to continental residency, and 5.8% was among meta-populations (Table S7).

With an increasing number of Y-STRs included in a marker set, the genetic distances between meta-populations decreased monotonical. However, the Yfiler panel was exceptional in this regard in that it yielded smaller distances than PPY23 for pairs of African and non-African meta-populations, but larger distances than PPY12 for pairs of non-African meta-populations (Table 4). Nevertheless, corresponding to the general trend, the proportion of variation both within populations and within meta-populations increased with increasing marker number, while the variation among populations decreased (Table S7). All covariance components associated with the different levels of continental groupings were significant (*p* < 10^−4^) for all marker sets (data not shown).

**Fig. 6:**
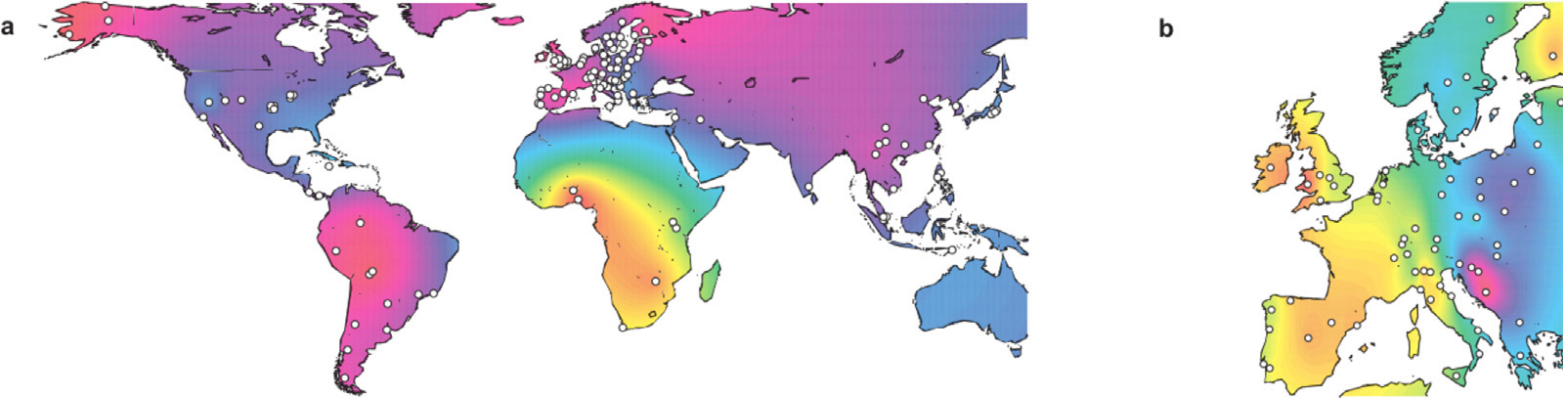
Population structure revealed by PPY23. Interpolated maps depict the first MDS components of pairwise *R*_ST_ values for PPY23. (a) Two-dimensional MDS analysis for the whole data set (129 populations); (b) four-dimensional MDS analysis for populations of European residency and ancestry alone (68 populations). Sample locations are marked in white. Color coding is on an arbitrary rainbow scale that assigns yellow and magenta to the opposite ends of the scale.

Multidimensional scaling (MDS) analysis was performed based upon linearized *R*_ST_, separately for the five marker sets, considering either all 129 populations or the 68 populations of European residency and ancestry alone. When assessed for the PPY23 marker panel, Kruskal's stress value showed a clear ‘elbow’ with increasing dimensionality in both population sets, pinpointing an optimal trade-off between explained variation and dimensionality. For the worldwide analysis, two MDS components were optimal with PPY23 whereas four components were deemed optimal for the Europeans-only analysis. Both solutions explained the haplotypic variation well, with *R*^2^ = 95.1% in the worldwide analysis and *R*^2^ = 99.2% in the Europeans-only analysis. For comparability, MDS analyses for other marker panels were carried out with two or four dimensions, respectively. Haplotypic variation among populations within continental groups was lower than between continental groups (Fig. S3). For all five marker sets, the first MDS component clearly separated the African populations from the non-African populations (Fig. 6a, Fig. S4). Moreover, MDS also confirmed the previously reported East–West separation in the Y-STR haplotype variation [32] in the European analysis (Fig. 6b, Fig. S5). Higher MDS components were strongly dependent upon the respective marker set (Figs. S4–S6) and lacked comparably clear population patterns.

Finally, the question was addressed of how closely related selected source and migrant populations might be in terms of their extant Y-STR haplotype spectra. A comparison between Han Chinese from Colorado (USA) and Han Chinese from Beijing, Chengdu (both China) and Singapore, respectively, yielded non-significant PPY23-based *R*_ST_ values (all ∼ 0) (Table S6). In strong contrast, African Americans from Illinois, the Southwest and the whole of the US were quite distant to Africans from Ibadan (Nigeria) (*R*_ST_ = 0.10, 0.13 and 0.09, respectively). Although likely not to represent the true source population, the distance between a group of Tamil from India and the Texan Gujarati population was as low as *R*_ST_ = 0.008, while the distance between the Tamils and a migrant Indian population in Singapore equalled 0.01. Finally, the distance between European Americans from Illinois, Utah and the whole USA on the one hand, and the Irish on the other was found to be consistently small (*R*_ST_ = 0.01, 0.04 and 0.02, respectively). A similar trend applied to other European source populations and to European migrant populations in South America. Thus, Argentineans of European ancestry from Buenos Aires, Formosa, Mendoza and Neuquen showed virtually zero genetic distance to Spaniards from Galicia (all three pairwise *R*_ST_ ∼ 0).

## 4 Discussion

In this study, by far the largest collection of Y-chromosomal STR haplotypes worldwide, genotyped with the PowerPlex^®^Y23 kit (PPY23; Promega Corporation) were compiled and analyzed. As expected, PPY23 provided higher discriminatory power for forensic purposes than other marker sets in our data. Remarkably, in almost one third of the populations studied, each sample could be identified unambiguously because all haplotypes in the population were unique. Most of the non-unique haplotypes were detected in populations that either passed through a recent bottleneck (e.g. Finland [33]) or that have a high reported degree of endogamy (e.g. Alaskan Natives and Kenyan Maasai). The higher number of unique haplotypes arising with PPY23 is a result of the larger number of markers in the kit and the preferential choice of markers with a higher discriminatory power. In particular, among the five Y-STRs with the highest diversity in our study, both globally and in all meta-populations, three (DYS481, DYS570 and DYS576) were specific to PPY23.

The practical utility of highly polymorphic Y-chromosomal profiles, for example, in biological stain analysis results from the greatly decreased chance of coincidental matches among different individuals. In the case of non-identity, exclusion becomes overwhelmingly likely. On the other hand, use of the PPY23 kit in kinship analysis or familial searching will render these practices increasingly complex because even close relatives may exhibit one or more mismatches, particularly at loci with high mutation rates. For these applications, there should be mandatory use of likelihood-based approaches that take allele frequencies, mutation rates and the presumed degree of relatedness properly into account [34].

The performance of forensic analysis with degraded DNA has also improved with the advent of PPY23. Typically, only partial DNA profiles can be generated from degraded DNA, with a pronounced dropout of longer amplicons. Compared to Yfiler, the short haplotypes of PPY23 (i.e. those comprising the eight markers with amplicons <220 bp) were much more variable. This difference is clearly due to the high mutation rates of four of the six markers specific to PPY23 selected for a short amplicon length. Thus, it is likely that the PPY23 kit will greatly improve the analysis of aged or otherwise damaged DNA samples.

The present study revealed a considerable number of null and duplicated alleles that were caused either by non-allelic homologous recombination between paralogous DNA sequences [35] or – in the case of nulls – by deletions or primer site mutations [36]. Compared to Yfiler, the PPY23 allelic ladder has been enriched with new length variants to accommodate the various intermediate alleles that were observed as well.

Previous population genetic analyses consistently revealed that Y-chromosomal haplotypes have a highly non-uniform geographical distribution characterized by less variation within, and more variation between, population groups than autosomal markers [37]. This difference has been explained by (i) the smaller effective population size of Y chromosomes causing stronger genetic drift, and (ii) haplotype clustering due to widespread patrilocality. Therefore, population structure, will be more pronounced in Y-chromosomal genetic databases and must be taken into account when database counts are used to quantify the evidential value of matches in forensic casework [38]. It has been shown, however, that so-called meta-populations may be constructed for Y-STRs that have low haplotypic variation among population groups within a meta-population, but large variation between meta-populations [39]. If necessary, such meta-populations can be defined ab initio using geography as a proxy of genetic relatedness, or by taking ethnic or linguistic data into account.

For all five forensic marker sets studied here, samples of African ancestry were clearly separated genetically from all other continental meta-populations. Pairwise genetic distances, measured by *R*_ST_, between Africa and the four non-African meta-populations were of similar magnitude. These results confirm a previous study of 40,669 haplotypes from 339 populations typed only for the nine markers of the MHT panel [39]. Moreover, genetic distances between non-African meta-populations were comparatively small. While North and South America still differed to some degree in the first MDS component, Eastern and Western Asia showed notable differences only in the second component. However, since the study here lacked samples from large parts of Northern and Central Asia, reasonable inference about the population structure in Asia as a whole was not possible.

Europe was the most intensively sampled continent in the present study and made up ∼60% of the overall sample size. A separate MDS analysis of samples of European residency and ancestry recapitulated the outcome of previous studies with smaller marker sets [32,40]. In particular, a clear East–West divide became evident in the first component of the MDS analysis for all five forensic marker sets. Finland and some regions of the Balkans (Croatia, Bosnia–Herzegovina) showed consistently large differences to other European populations in the second MDS component.

It must be emphasized that this population genetic analysis was based upon marker sets that were designed for forensic purposes, and that shared several markers. That all five sets yielded a similar picture of the geographic distribution of Y-STR haplotypes may therefore indicate that, in terms of population structure, the effects of markers included in the MHT (which are common to all five sets) dominate those of more mutable markers, such as PPY23-specific STRs DYS576, DYS570 and DYS481. Indeed, it has been shown recently that haplotypes comprising only rapidly mutating markers lack strong signals of population history (Ballantyne et al., submitted for publication).

In summary, the recently introduced PowerPlex^®^Y23 system provides unprecedented discriminatory power for forensic applications but at the same time shows similar patterns of population structure as established forensic marker sets. These patterns coincide well with groupings according to prior information on geographical and ethnic origin. In some cases, relatively large genetic distances were found for pairs of migrant and potential source populations, such as African Americans and autochthonous Africans. For forensic casework involving these populations, separate reference databases need to be established and used. On the other hand, populations showing small genetic distances, such as Western or Eastern Europeans, Arabs from Iraq and Lebanon or Mestizos from Peru and Bolivia may be merged into meta-populations for the purpose of reference databases. The annotated PPY23 data used in this study have been fully integrated into the YHRD database as of October 2013 (release 45, www.yhrd.org).

## Figures and Tables

**Table 1:** Number of distinct haplotypes for the PPY23 marker panel overall and for five groups defined by continental residency.

	Overall	Africa	Asia	Europe	Latin America	North America
	19,630 haplotypes^f^	445 haplotypes^f^	3458 haplotypes^f^	11,968 haplotypes^f^	1183 haplotypes^f^	2576 haplotypes^f^
	129 populations	6 populations	22 populations	72 populations	14 populations	15 populations
*n* = 1 (unique)	18,237 (92.9%)	337 (75.7%)	3293 (95.2%)	11,185 (93.5%)	1094 (92.5%)	2378 (92.3%)
*n* = 2	531	27	65	314	38	67
*n* = 3	64	6	9	32	3	12
*n* = 4	16	4	2	7	1	3
*n* = 5	6^a^	1		3		2
*n* = 6	2^b^	–		1		1
*n* = 7	2^c^	1		–		
*n* = 8	1^d^	1		–		
*n* = 9	–			–		
*n* = 10	–			1		
*n* = 11	1^e^					

HD	0.999995	0.998704	0.999983	0.999992	0.999924	0.999953
MP	5.63 × 10^−5^	3.54 × 10^−3^	3.07 × 10^−4^	9.11 × 10^−5^	9.21 × 10^−4^	4.35 × 10^−4^
MP as 1 in …	17,760	283	3262	10,975	1086	2298
# Distinct haplotypes	18,860	377	3369	11,543	1136	2463
# Population specific haplotypes	–	376	3367	11,516	1127	2446
DC	0.9608	0.8472	0.9743	0.9645	0.9603	0.9561

HD, haplotype diversity; MP, match probability; DC, discrimination capacity. Number and origin of individuals sharing a distinct haplotype.

a Northern Alaska [Inupiat] 5; Northern Alaska [Inupiat] 5; Krakow, Poland [Polish] 1; Netherlands [Dutch] 4; Finland [Finnish] 5; Estonia [Estonian] 1; Finland [Finnish] 3; Turku, Finland [Finnish] 1; Kinyawa, Kenya [Maasai] 5.

b Northern Alaska, USA [Inupiat] 6; Estonia [Estonian] 1; Finland [Finnish] 3; Western Alaska, USA [Yupik] 2.

c South Africa [Xhosa] 7; Baranya, Hungary [Romani] 2; Hungary [Hungarian] 2; Hungary [Romani] 2; USA [European American] 1.

d Kinyawa, Kenya [Maasai] 8.

e Central Alaska, USA [Athapaskan] 1; Estonia [Estonian] 1; Finland [Finnish] 6; Turku, Finland [Finnish] 3.

f # Haplotypes observed.

**Table 2:** Diversity values and forensic parameter estimates for five forensic marker panels.

# Haplotypes observed	Forensic marker set				
19,630 samples	MHT	SWGDAM	PPY12	Yfiler	PPY23^a^
	9 marker	11 marker	12 marker	17 marker	23 marker
*n* = 1 (unique)	6083 (31.0%)	8495 (43.3%)	9092 (46.3%)	15,263 (77.8%)	18,237 (92.9%)
*n* = 2	1131	1227	1260	1064	531
*n* = 3	435	436	416	256	64
*n* = 4	226	199	196	94	16
*n* = 5	114	101	106	63	6
*n* = 6	86	85	85	21	2
*n* = 7	63	51	50	12	2
*n* = 8	43	50	41	12	1
*n* = 9	29	29	34	9	–
*n* = 10	31	21	24	4	–
*n* = 11	22	24	28	5	1
*n* = 12	21	21	16	3	
*n* = 13	14	14	9	2	
*n* = 14	14	15	12	1	
*n* = 15	15	8	4	2	
*n* = 16	13	3	5	–	
*n* = 17	11	3	2	2	
*n* = 18	9	2	7	1	
*n* = 19	4	9	4	–	
*n* = 20	6	–	3	2	
*n* = 21	3	4	3	1	
*n* = 22	3	4	2	1	
*n* = 23	5	1	–	–	
*n* = 24	2	4	4	–	
*n* = 25	3	1	4	1	
*n* = 26	3	1	1	–	
*n* = 27	3	4	2	–	
n = 28	2	1	1	–	
*n* = 29	3	1	1	–	
*n* = 30	2	1	2	–	

*n* ∈ (30, 40]	13	11	7	1	
*n* ∈ (40, 50]	9	8	11		
*n* ∈ (50, 60]	5	4	6		
*n* ∈ (60, 70]	5	7	3		
n ∈ (70, 80]	5	1	–		
*n* ∈ (80, 90]	2	–	–		
*n* ∈ (90, 100]	2	2	1		

*n* = 106	2	–	–		
*n* = 118	1	–	1		
*n* = 126	–	–	1		
*n* = 135	–	1	–		
*n* = 143	–	1	–		
*n* = 170	–	–	1		
*n* = 182	1	–	–		
*n* = 203	–	1	–		
*n* = 215	1	–	–		
*n* = 242	–	–	1		
*n* = 268	1	–			
*n* = 290	–	1			
*n* = 331	1				
*n* = 515	1				

HD	0.998033	0.999199	0.999394	0.999962	0.999995
MP	2.02 × 10^−3^	8.52 × 10^−4^	6.57 × 10^−4^	8.94 × 10^−5^	5.63 × 10^−5^
MP as 1 in …	496	1175	1522	11,189	17,760
# Distinct haplotypes	8448	10,852	11,446	16,820	18,860
DC	0.4304	0.5528	0.5831	0.8569	0.9608

a Diversity values and forensic parameter estimates for the PPY23 panel were taken from Table 1. MHT, minimal haplotype; SWGDAM, Scientific Working Group for DNA Analysis Methods; PPY12, PowerPlex^®^Y12; Yfiler, Yfiler^®^kit; PPY23, PowerPlex^®^Y23; HD, haplotype diversity; MP, match probability; DC, discrimination capacity.

**Table 3:** Forensic parameter estimates for short STRs <220 bp included in the Yfiler and PPY23 marker panels.

	Yfiler short^a^	PPY23 short^b^
HD	0.998569	0.999695
MP	1.48 × 10^−3^	3.56 × 10^−4^
MP as 1 in …	675	2809
# distinct haplotypes	6418	11,702
DC	0.3269	0.5961

HD, haplotype diversity; MP, match probability; DC, discrimination capacity

a Includes markers DYS456, **DYS389I**, **DYS458**, DYS19, **DYS393**, **DYS391**, GATAH4, DYS437.

b Includes markers DYS576, **DYS389I**, **DYS391**, DYS481, DYS570, DYS635, **DYS393**, **DYS458.**

**Table 4:** Pairwise *R*_ST_ value estimates (below the diagonal) and corresponding *p* value (above the diagonal) between meta-populations defined by continental residency for five forensic marker panels.

Marker set	Continent	Africa	Asia	Europe	Latin America	North America
MHT	Africa		+	+	+	+
	Asia	0.33675		+	+	+
	Europe	0.31720	0.07390		+	+
	Latin America	0.31534	0.07258	0.04588		+
	North America	0.23944	0.05755	0.02525	0.01369	

SWGDAM	Africa		+	+	+	+
	Asia	0.29365		+	+	+
	Europe	0.25428	0.07710		+	+
	Latin America	0.27268	0.08041	0.03879		+
	North America	0.20579	0.06960	0.02477	0.01083	

PPY12	Africa		+	+	+	+
	Asia	0.28688		+	+	+
	Europe	0.25591	0.08023		+	+
	Latin America	0.26814	0.07821	0.03884		+
	North America	0.20601	0.06886	0.02465	0.01036	

Yfiler	Africa		+	+	+	+
	Asia	0.22740		+	+	+
	Europe	0.22823	0.10203		+	+
	Latin America	0.23016	0.08182	0.02695		+
	North America	0.1755	0.07394	0.01914	0.0081	

PPY23	Africa		+	+	+	+
	Asia	0.26082		+	+	+
	Europe	0.28376	0.06978		+	+
	Latin America	0.28108	0.05315	0.01653		+
	North America	0.21391	0.04687	0.01842	0.00895	

Pairwise genetic distances (*R*_ST_ value estimates, below the diagonal) were obtained by an analysis of molecular variance (AMOVA). Significance of genetic distances (above the diagonal) was tested by 10,000 permutations. All *p* values were <10^−4^ (highlighted by +). MHT, minimal haplotype; SWGDAM, Scientific Working Group for DNA Analysis Methods; PPY12, PowerPlex^®^Y12; Yfiler, Yfiler^®^kit; PPY23, PowerPlex^®^Y23.
