# Supplementary figures and images for: A global analysis of Y-chromosomal haplotype diversity for 23 STR loci
Source: Forensic Sci Int Genet. 2014 Sep;12(100):12–23. doi: 10.1016/j.fsigen.2014.04.008 (PMC4127773; doi:10.1016/j.fsigen.2014.04.008)

a

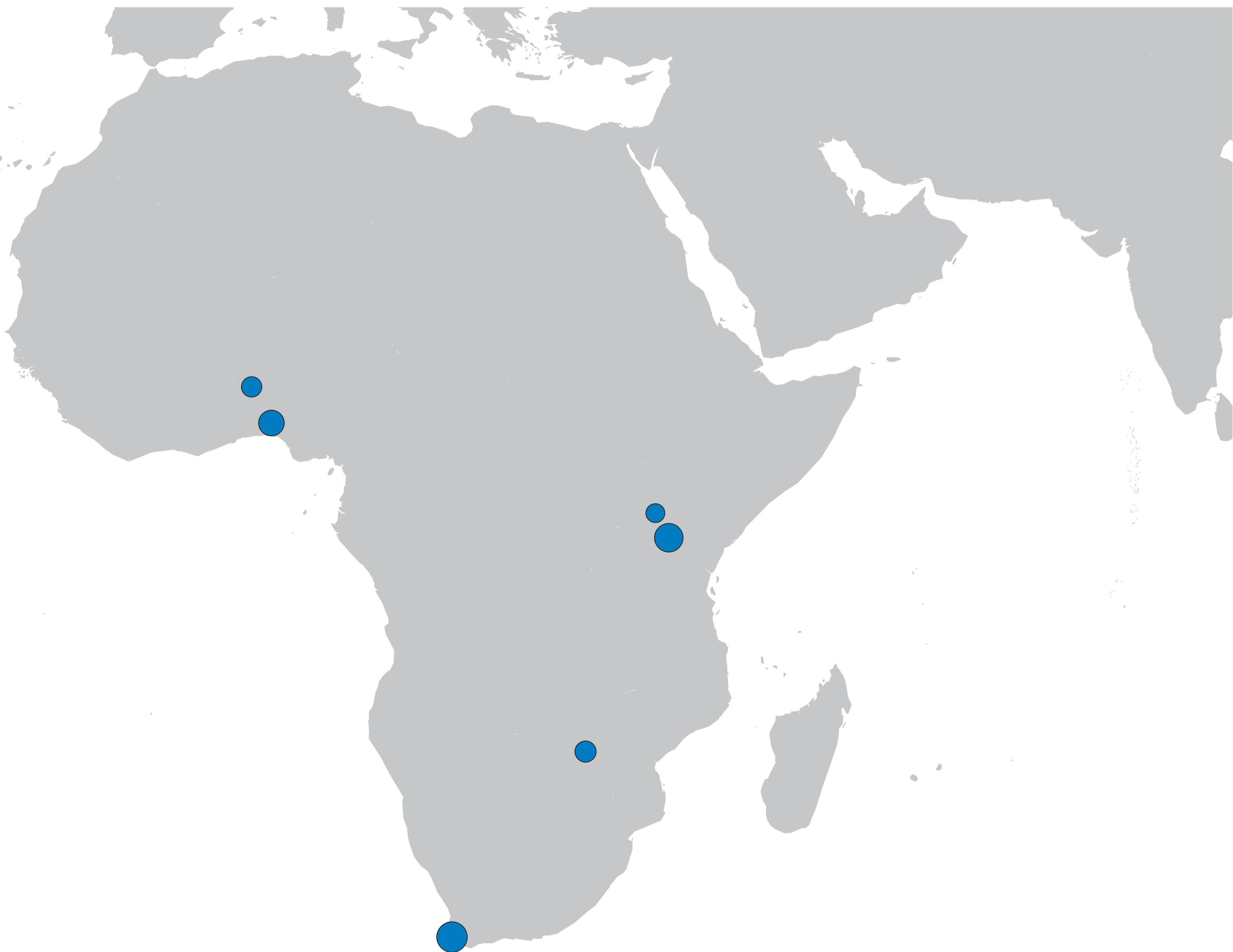

**b**

C

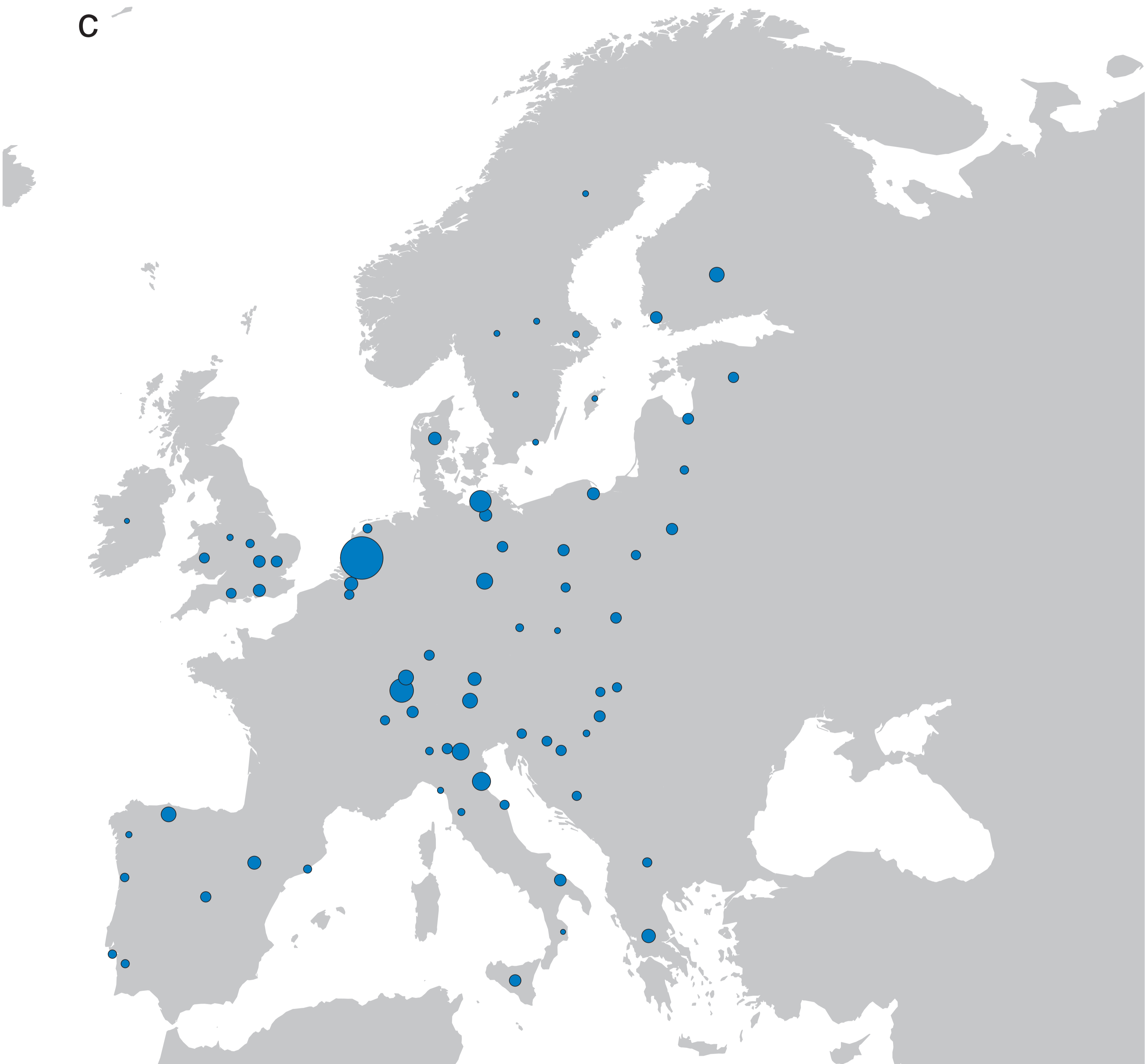

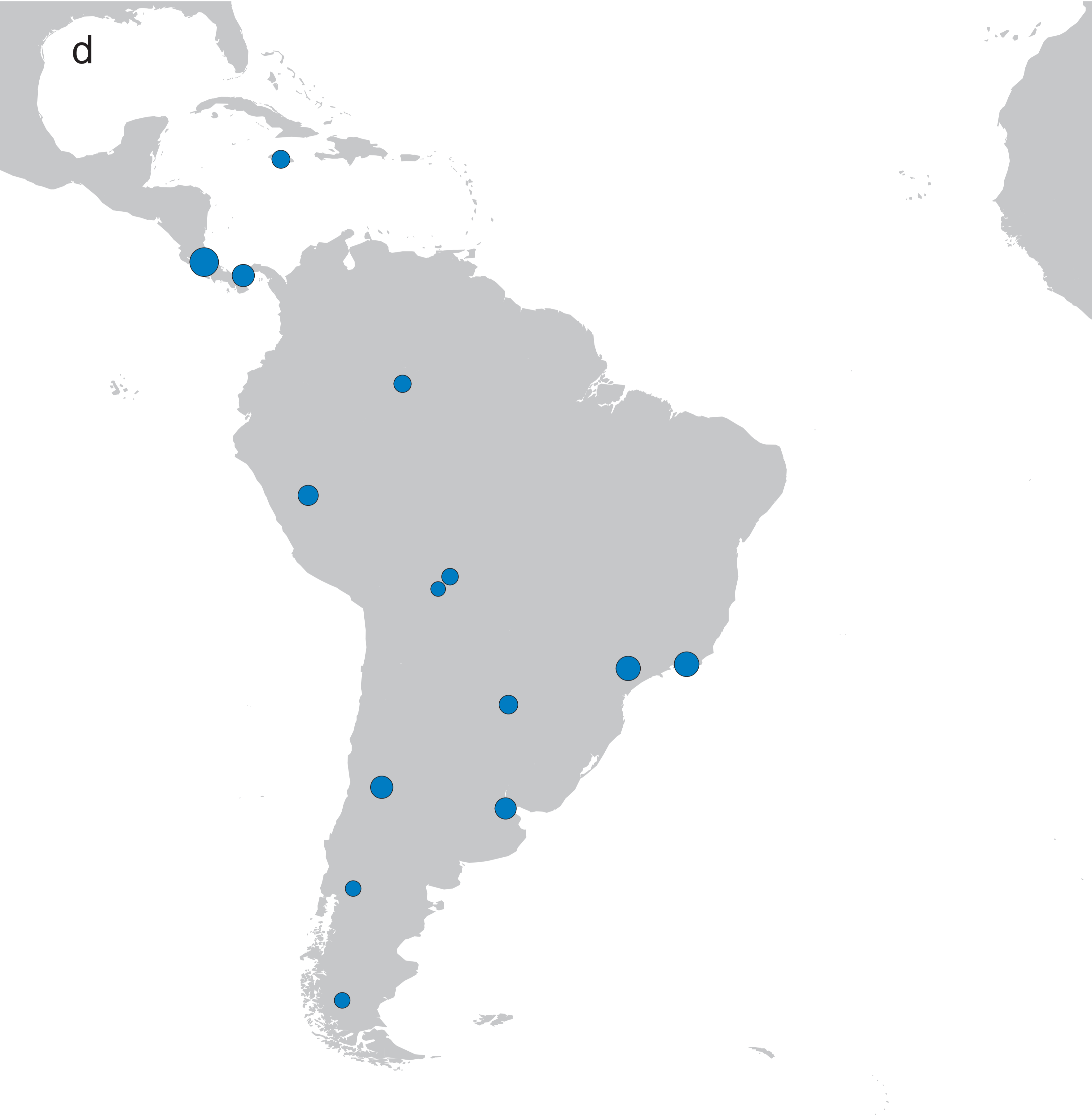

e

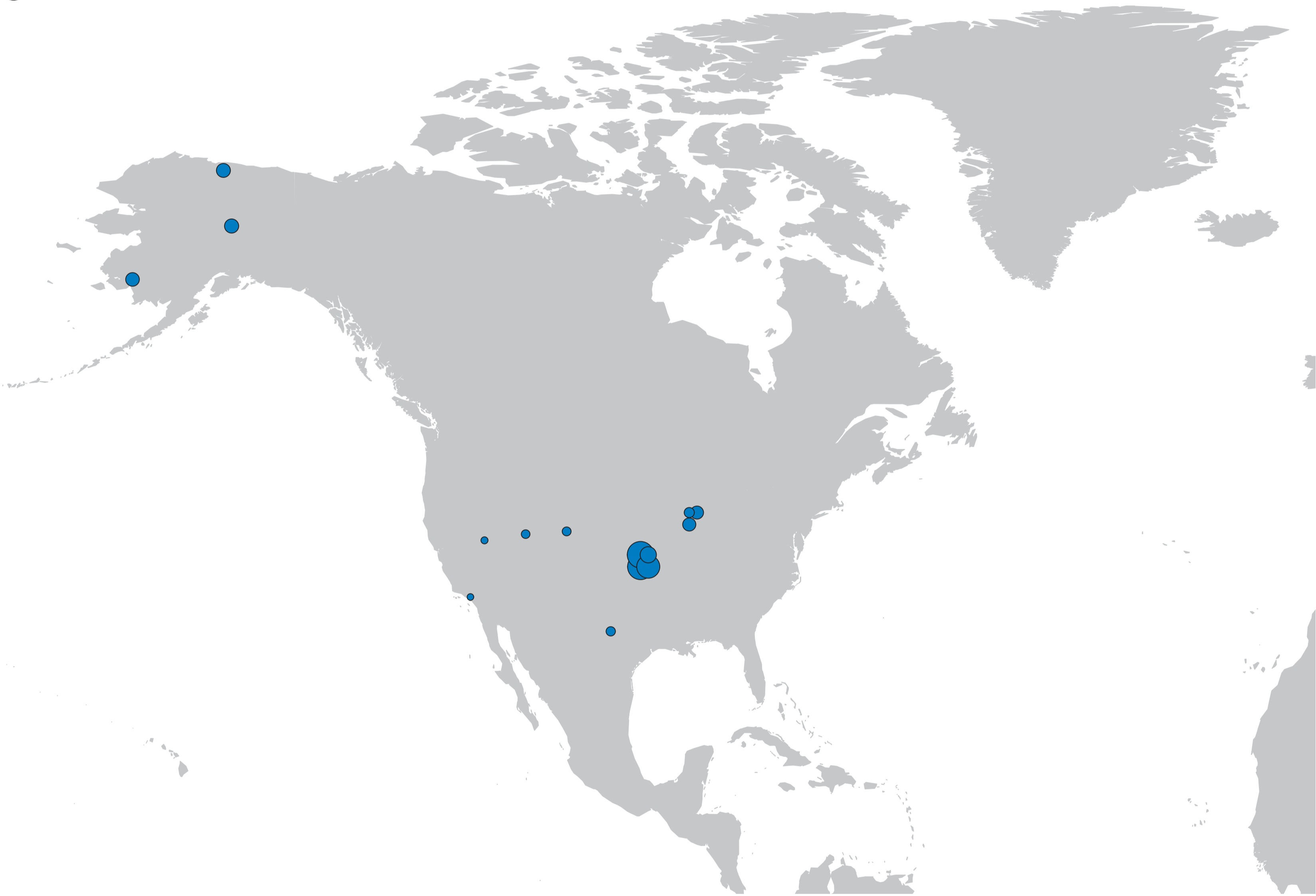

Supplement: Supplementary file 8 [file mmc8.pdf]

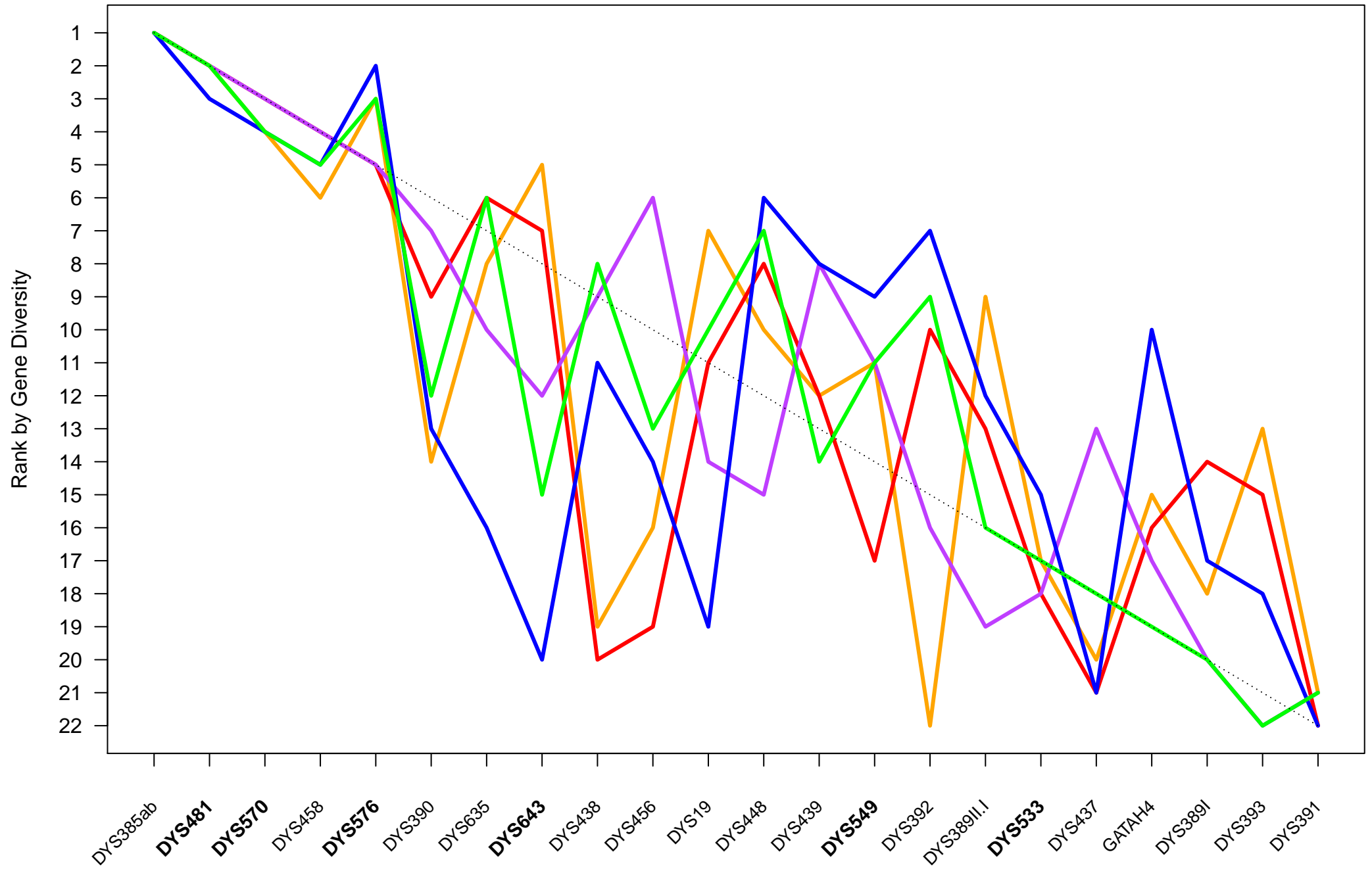

Supplement: Supplementary file 9 [file mmc9.pdf]

**a**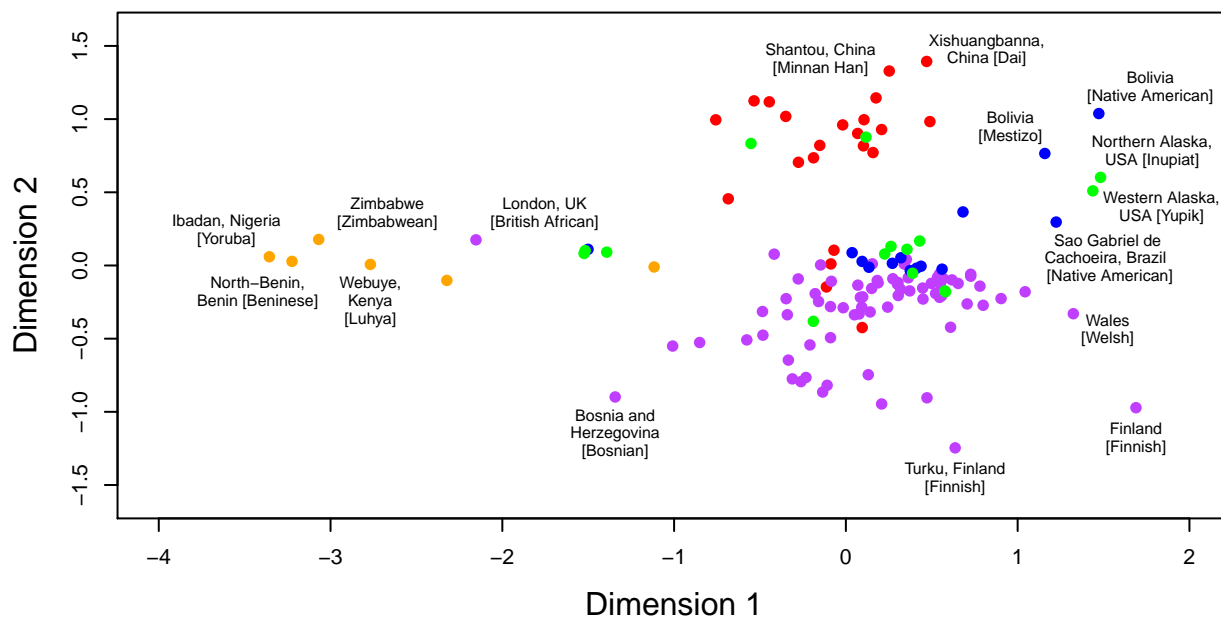**b**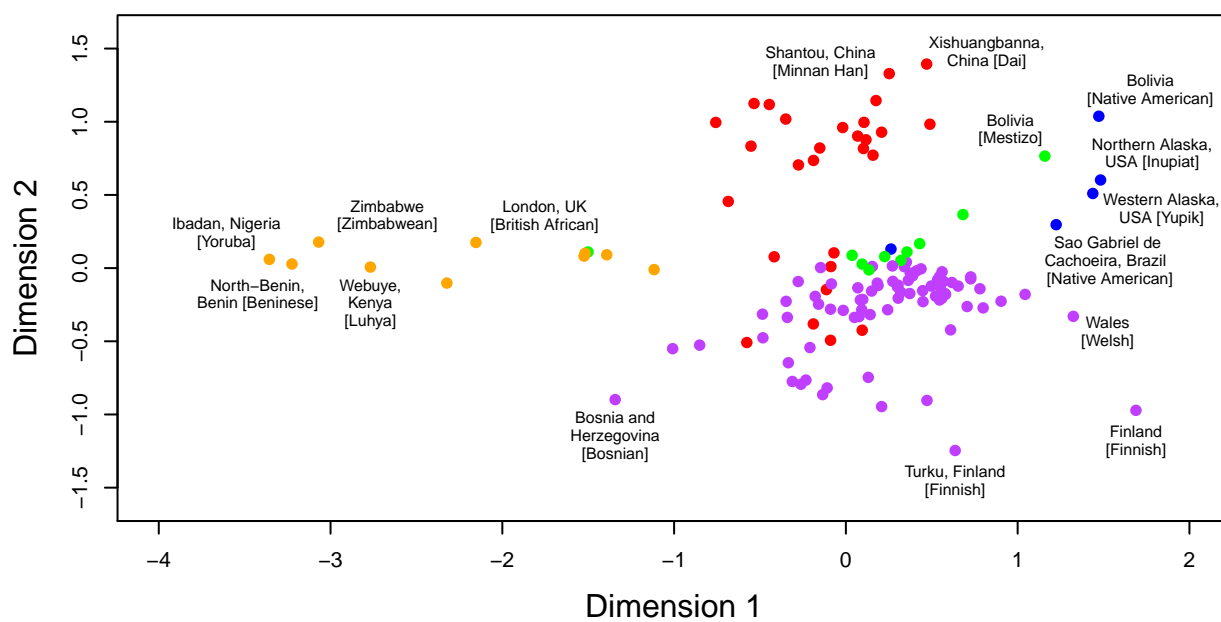

Supplement: Supplementary file 10 [file mmc10.pdf]

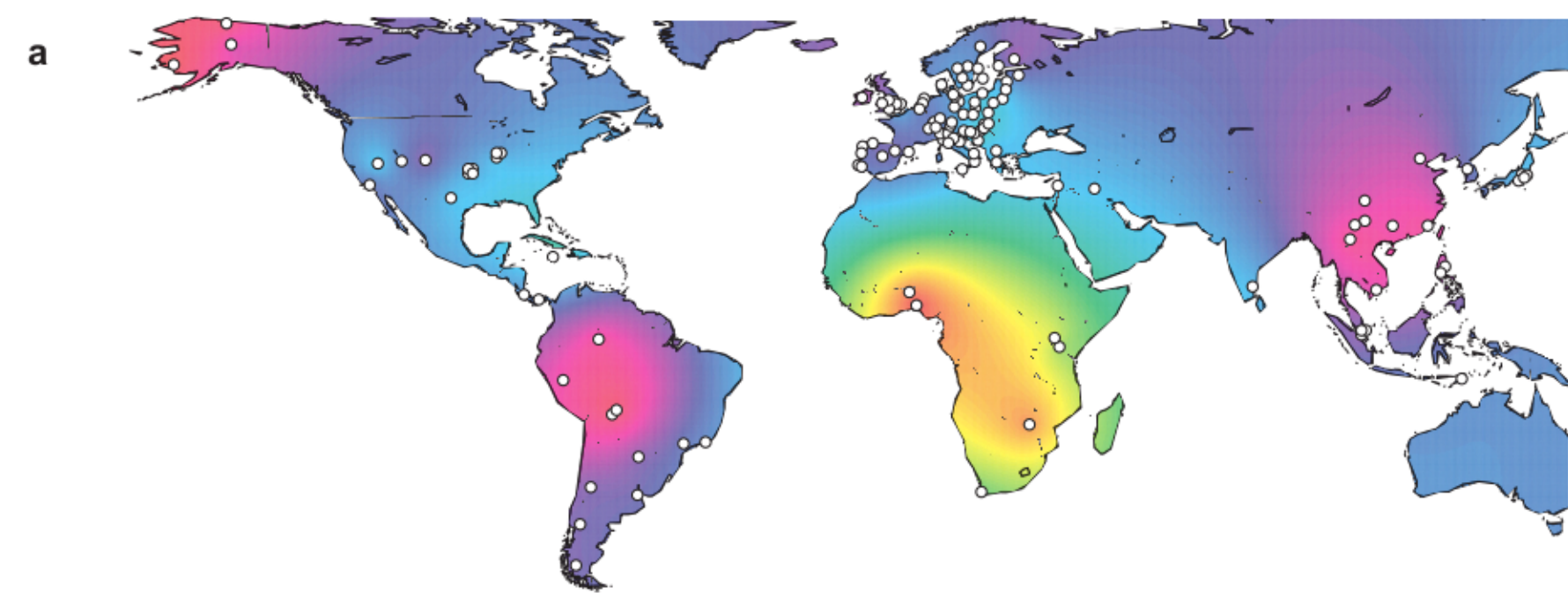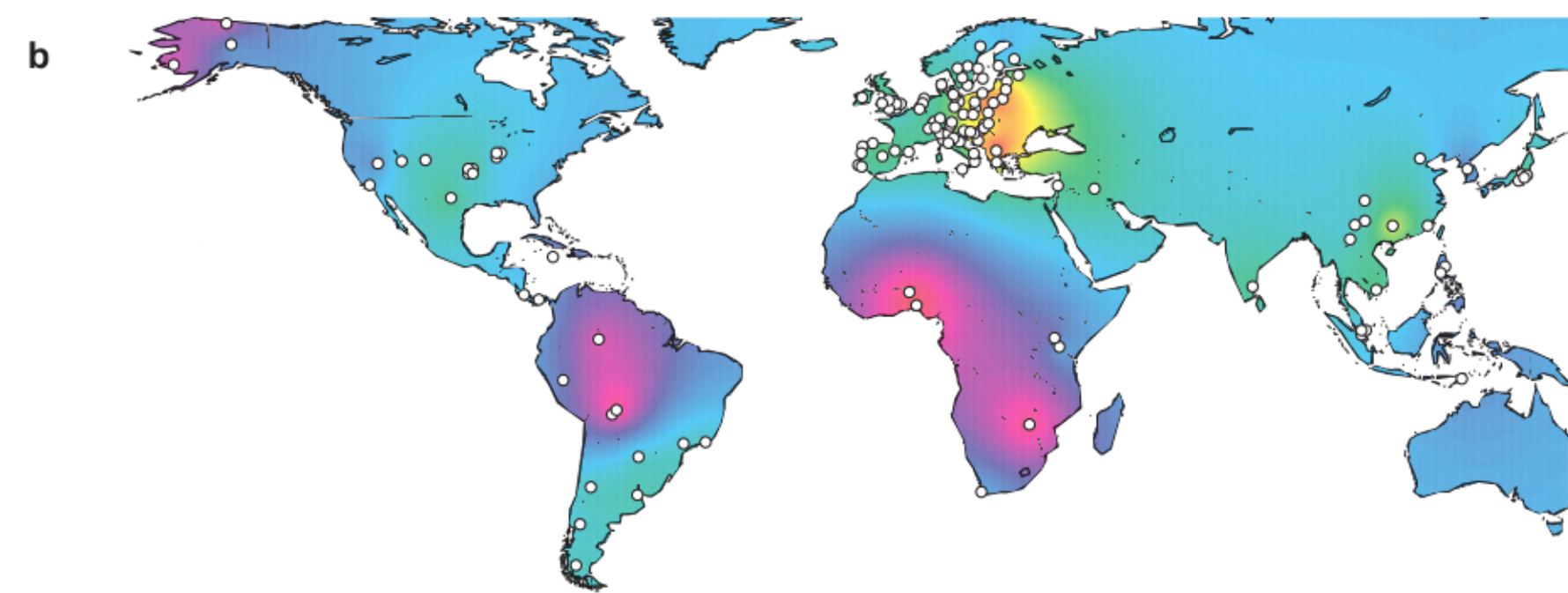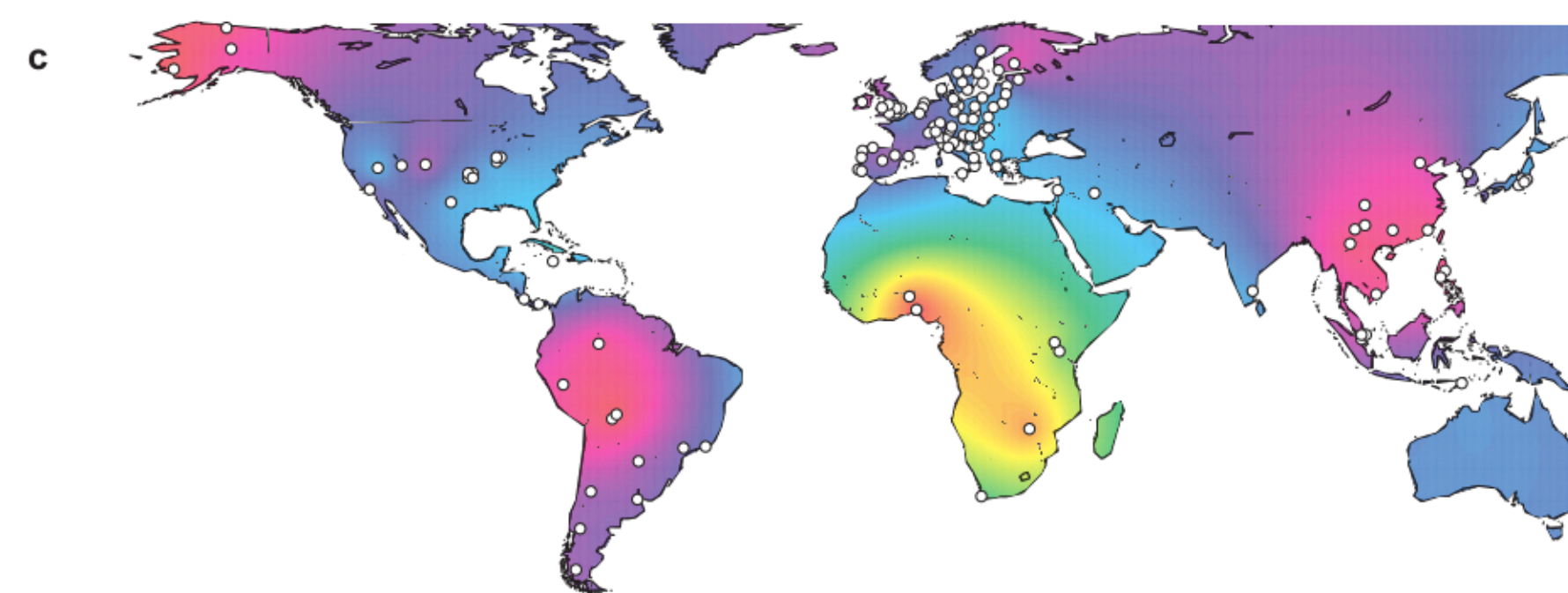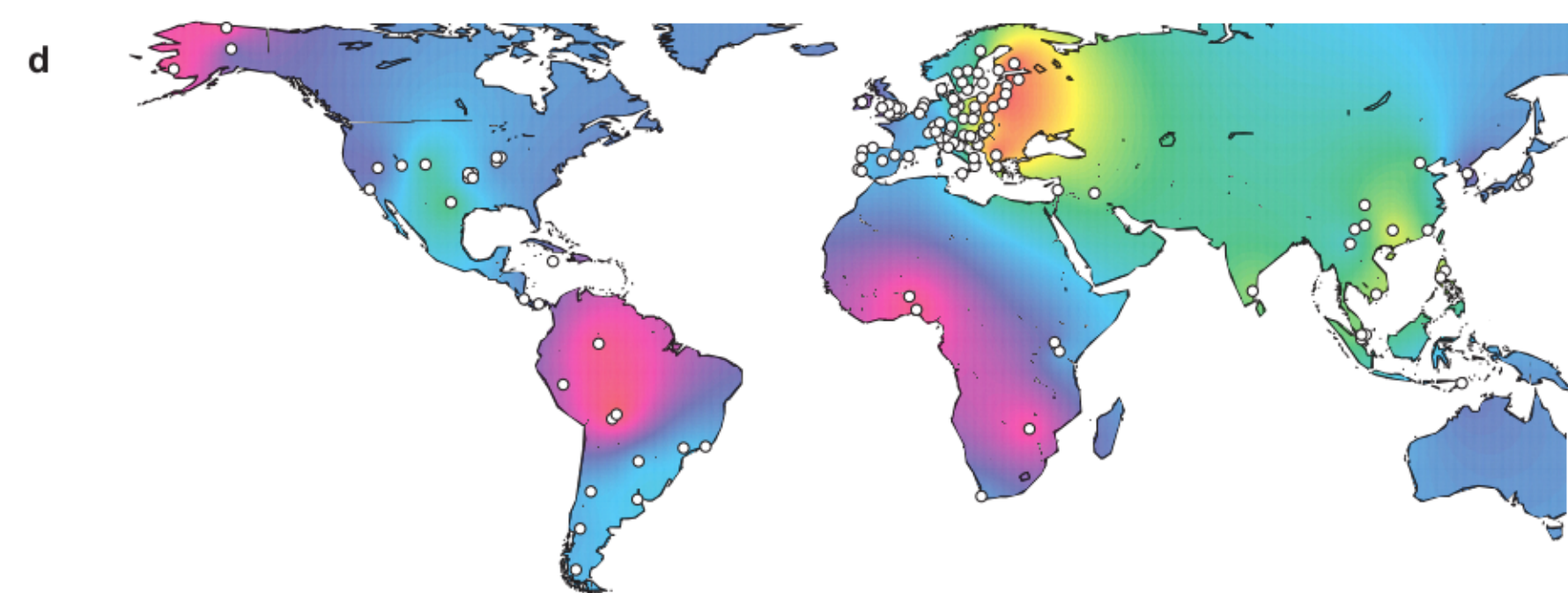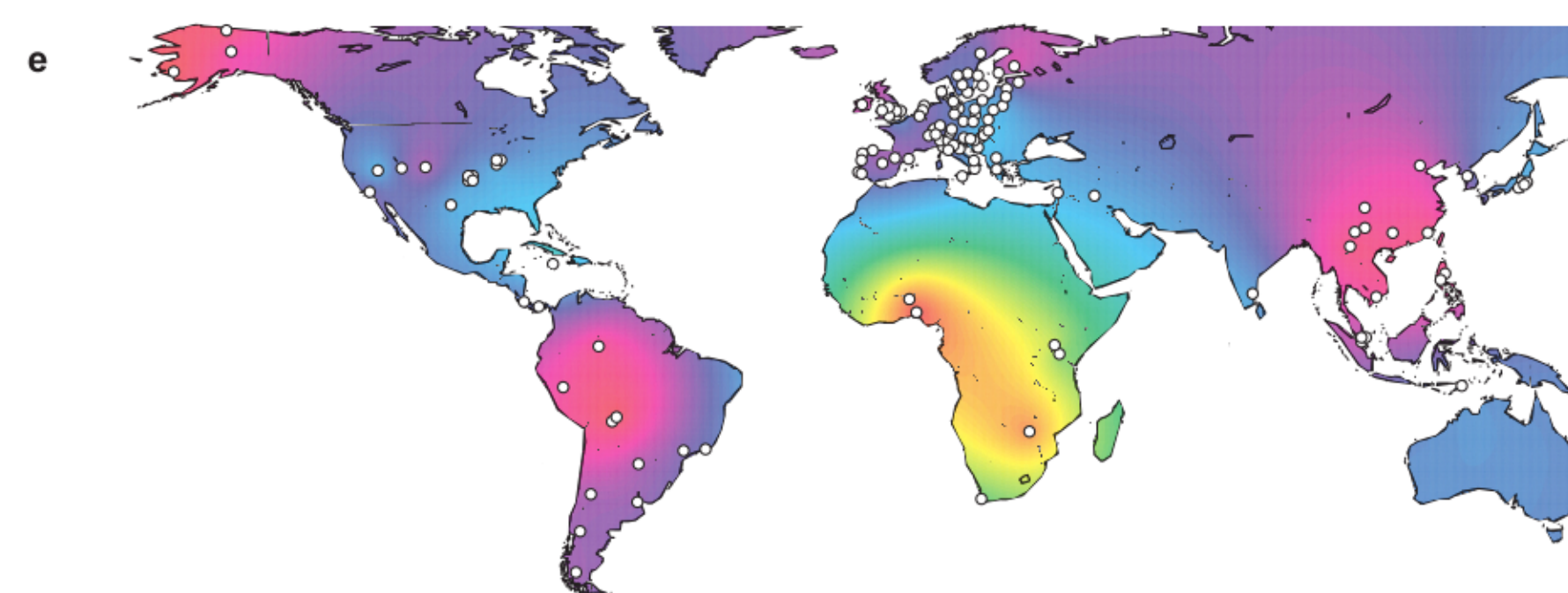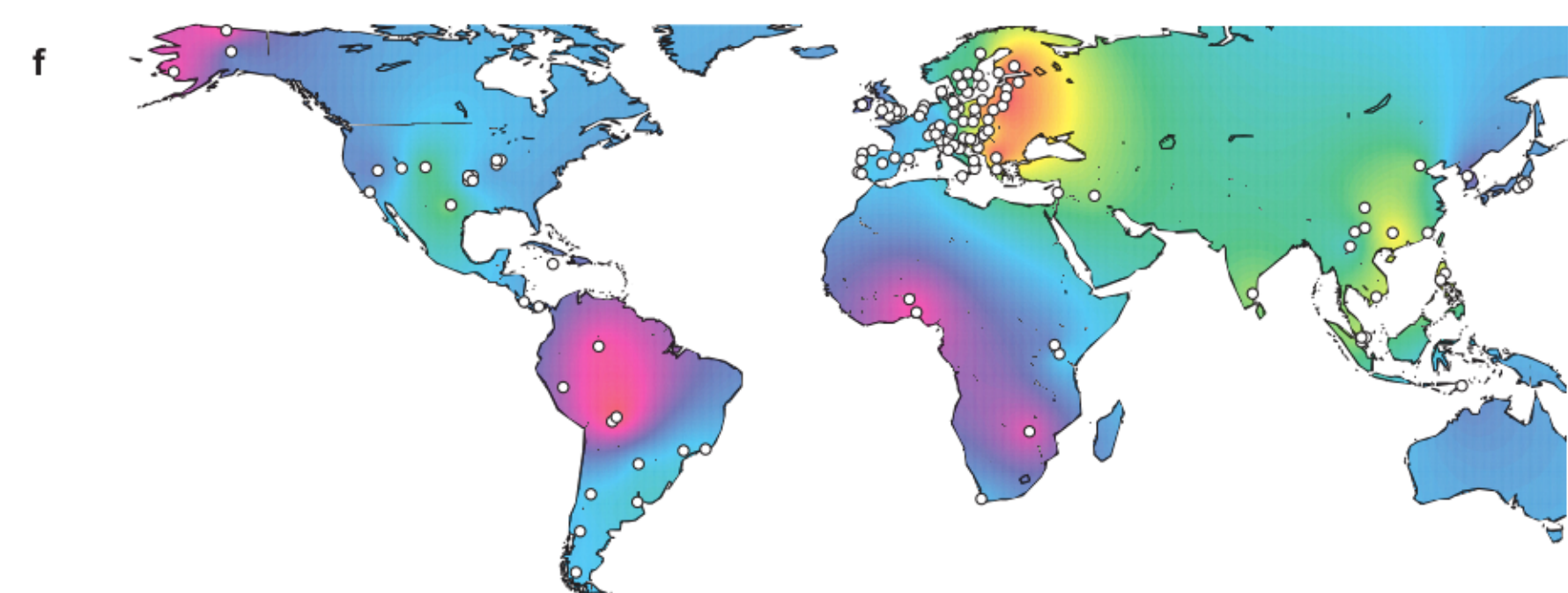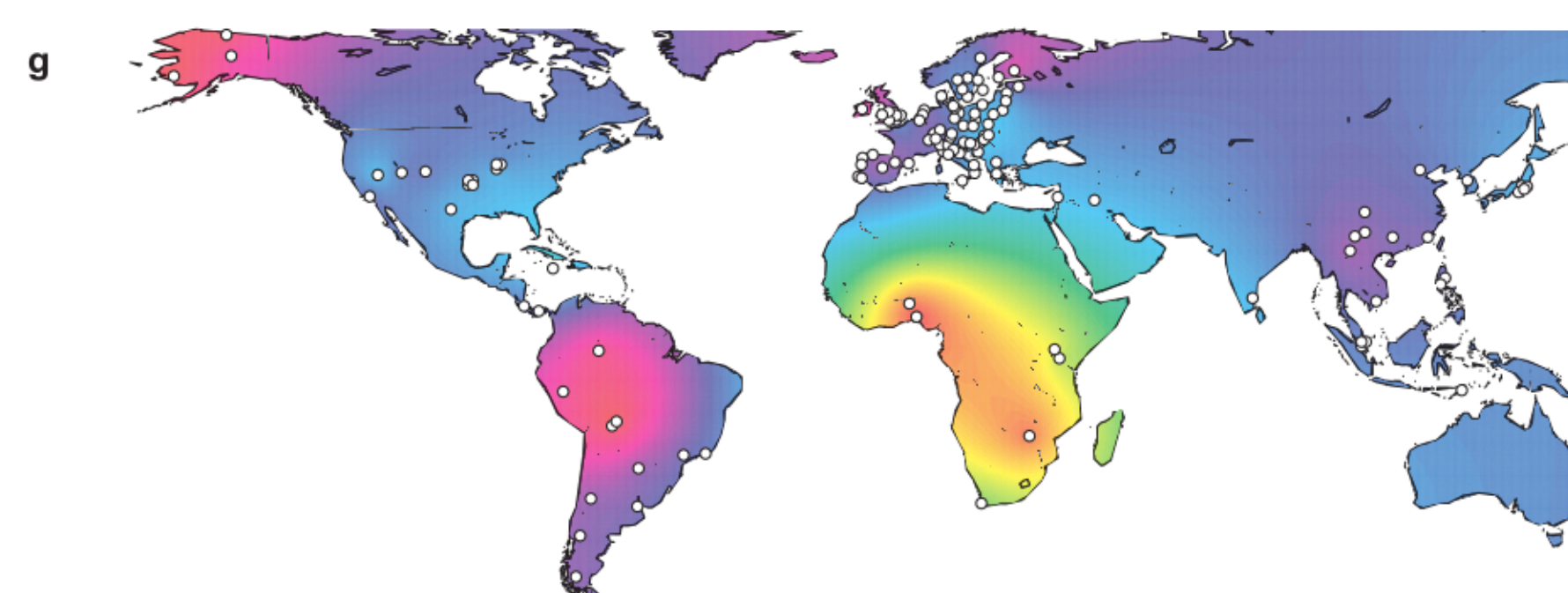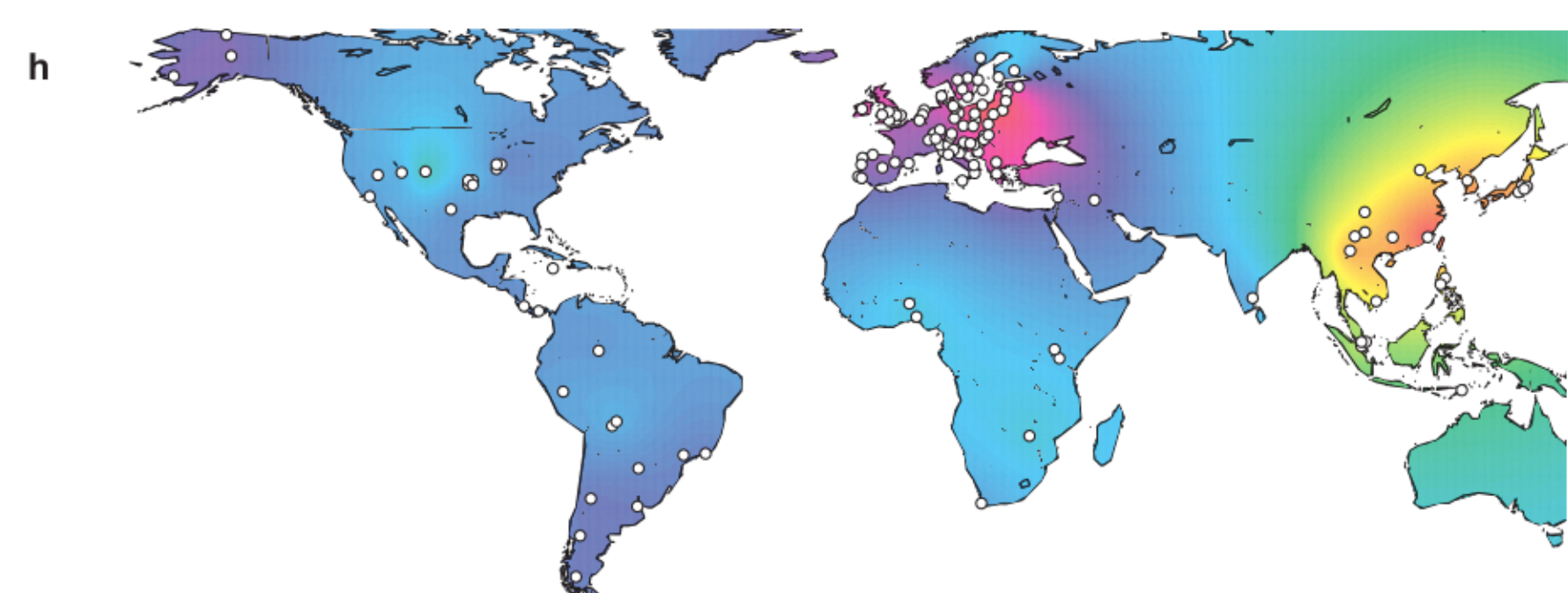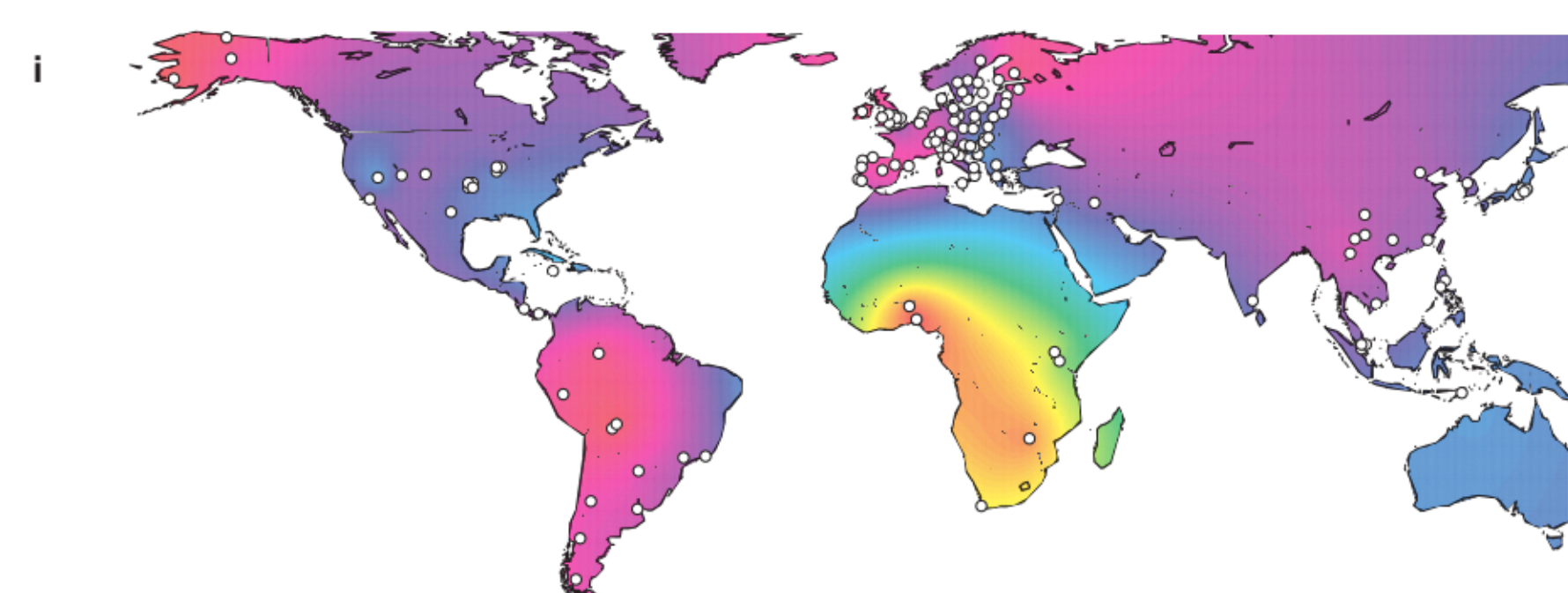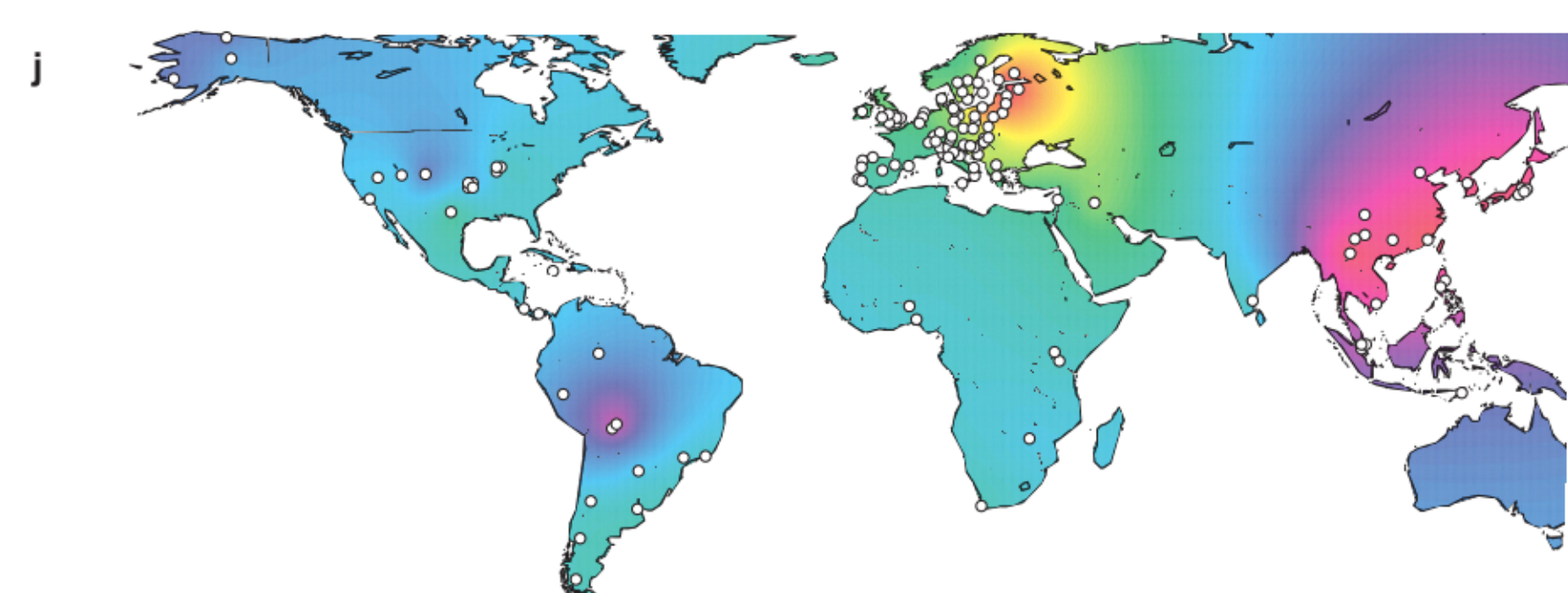

Supplement: Supplementary file 11 [file mmc11.pdf]

a

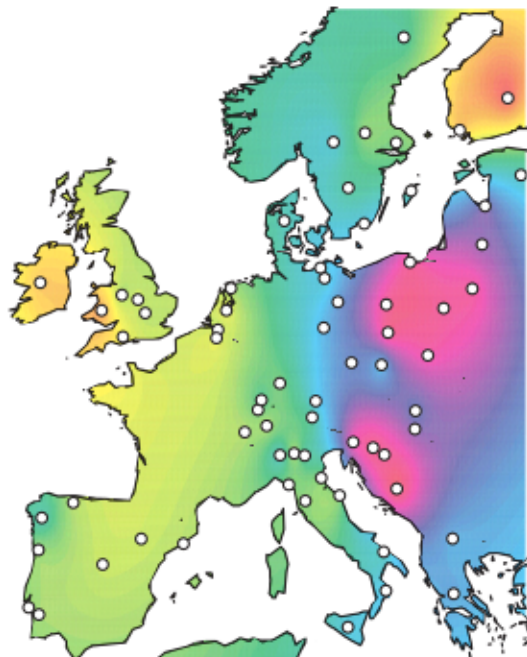

b

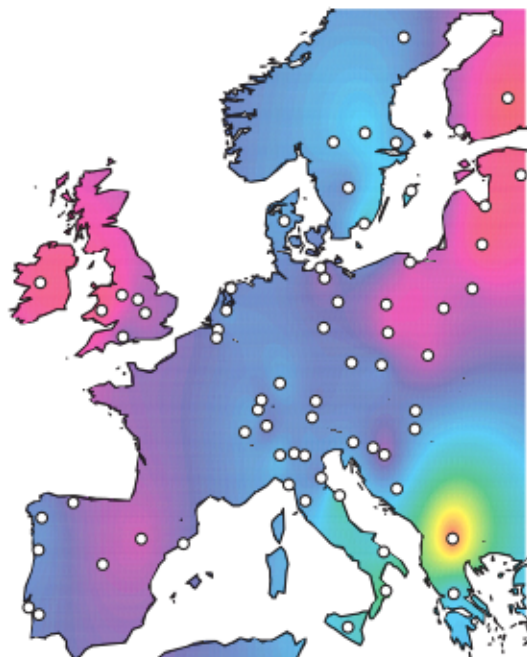

c

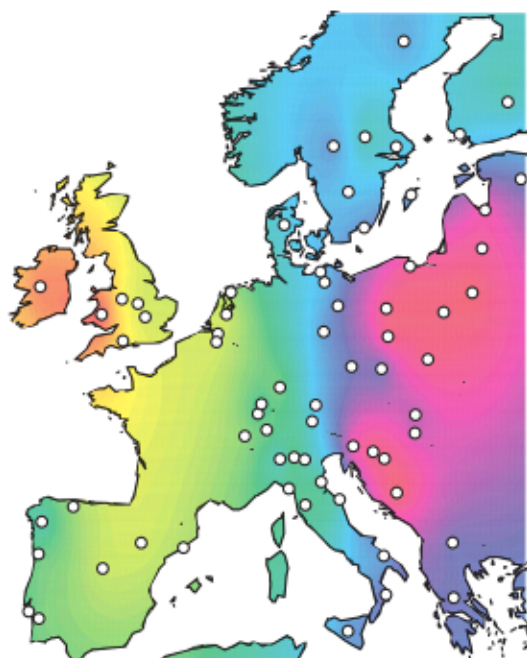

d

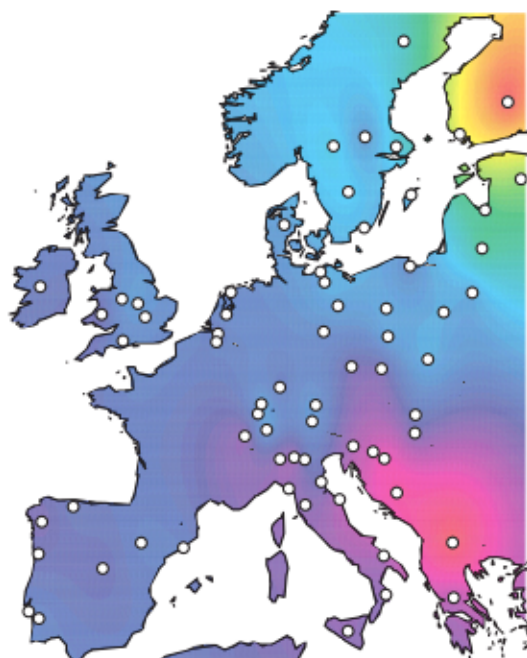

e

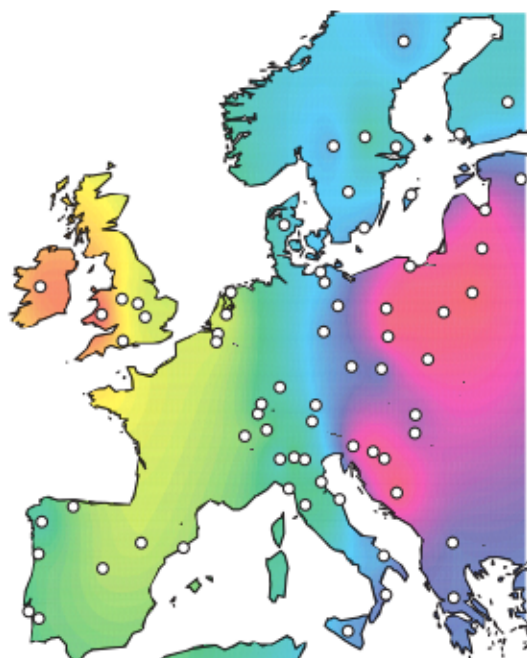

f

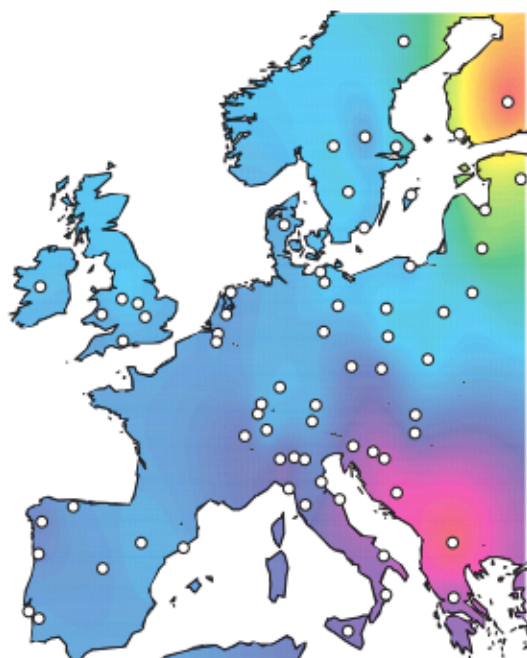

g

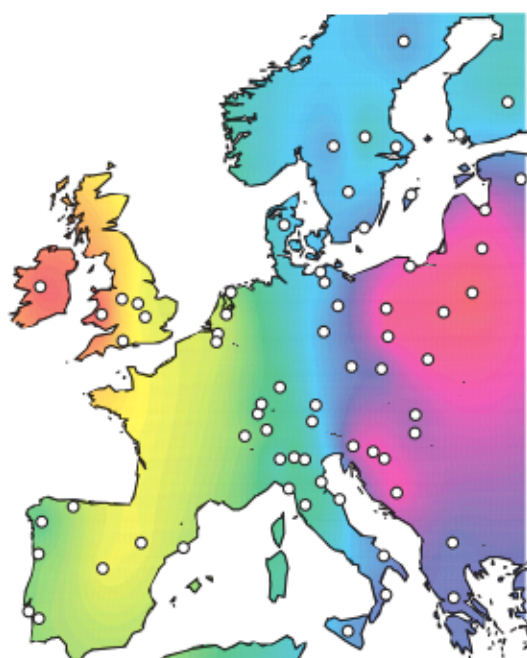

h

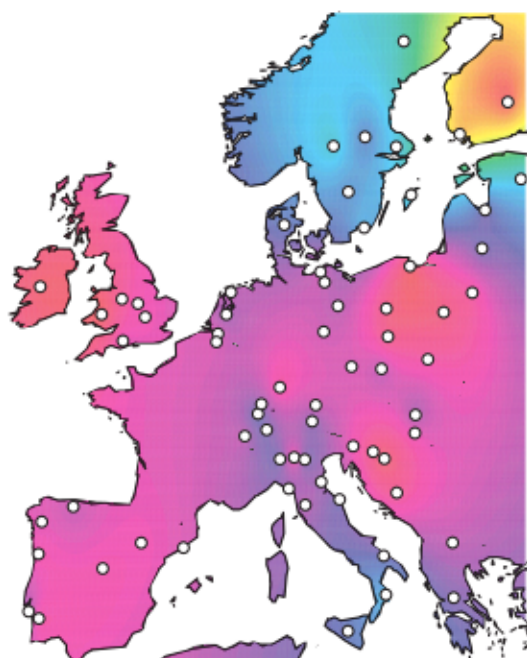

i

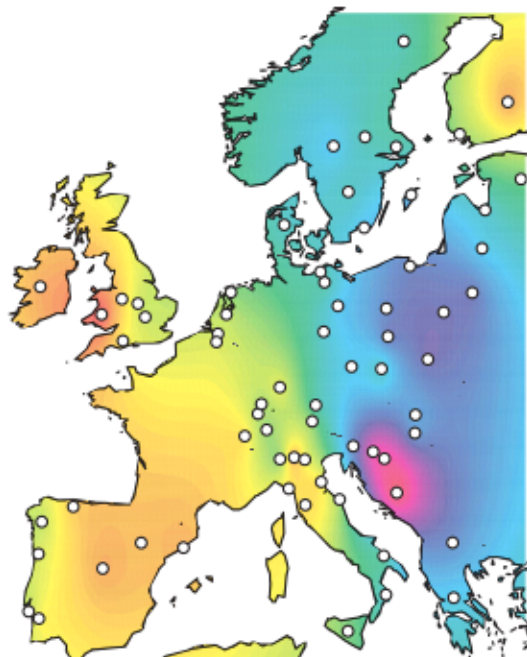

j

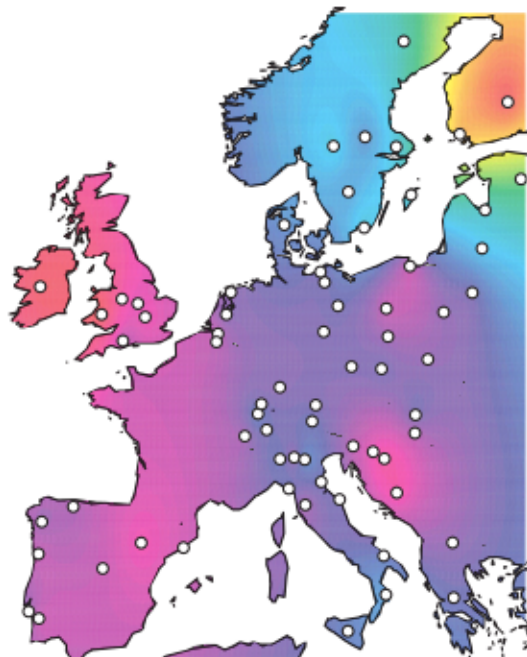

Supplement: Supplementary file 12 [file mmc12.pdf]

a

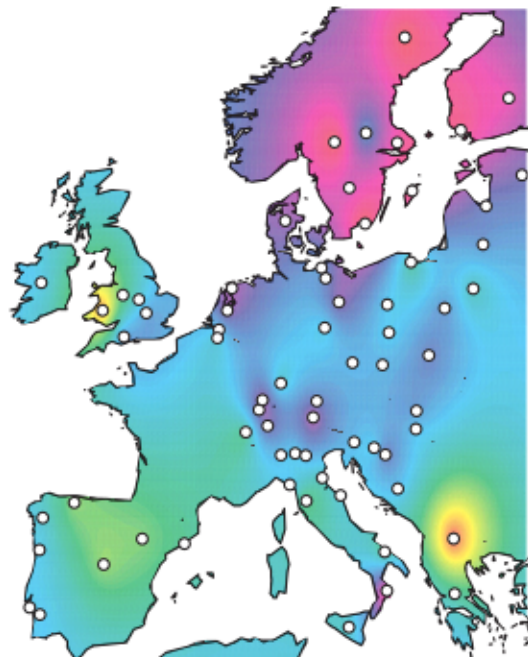

b

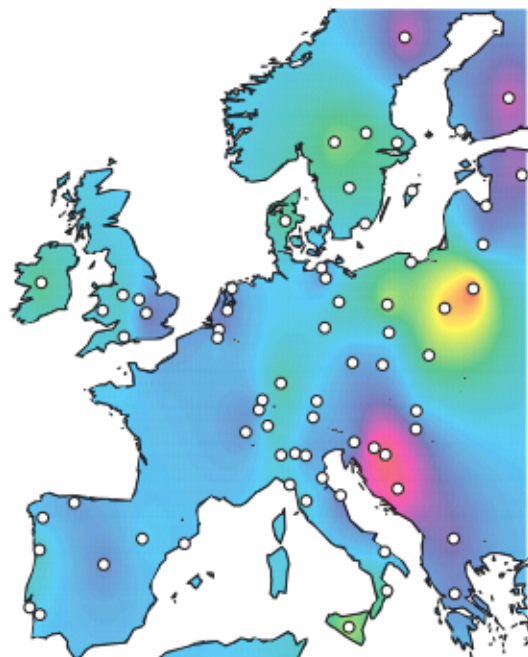

c

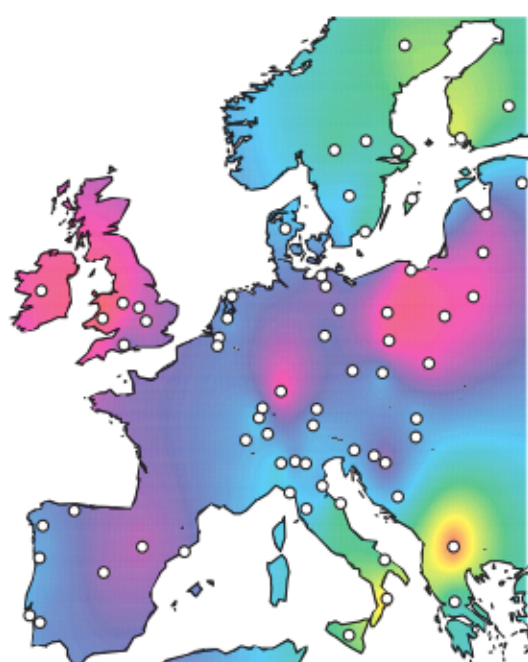

d

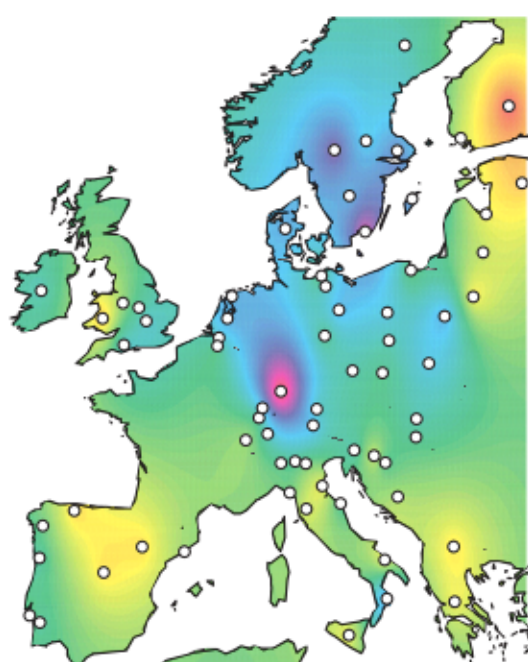

e

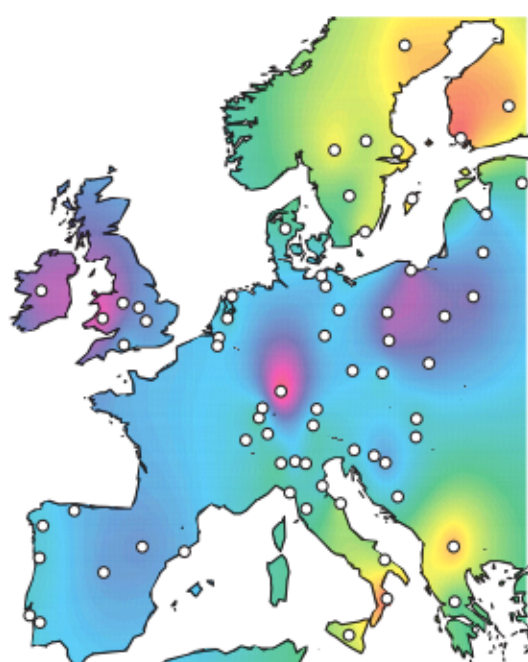

f

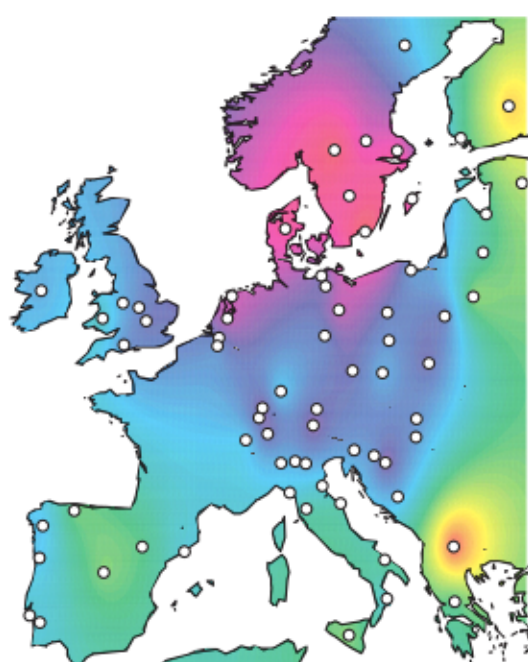

g

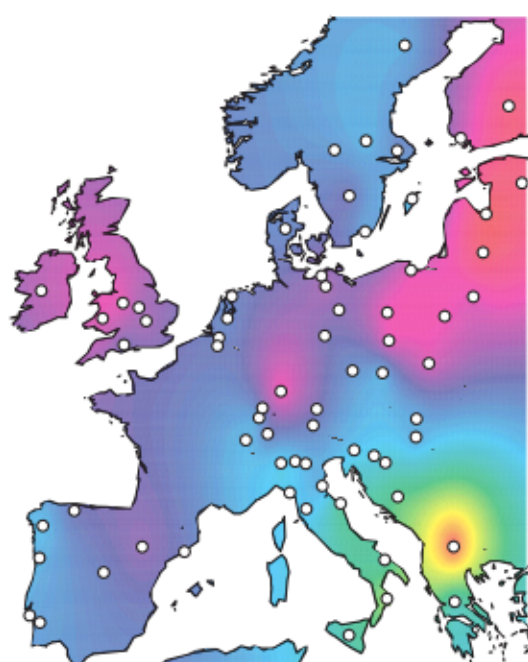

h

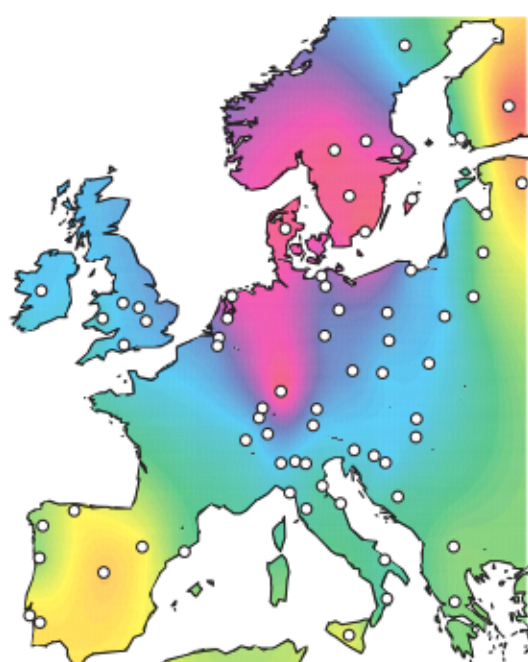

i

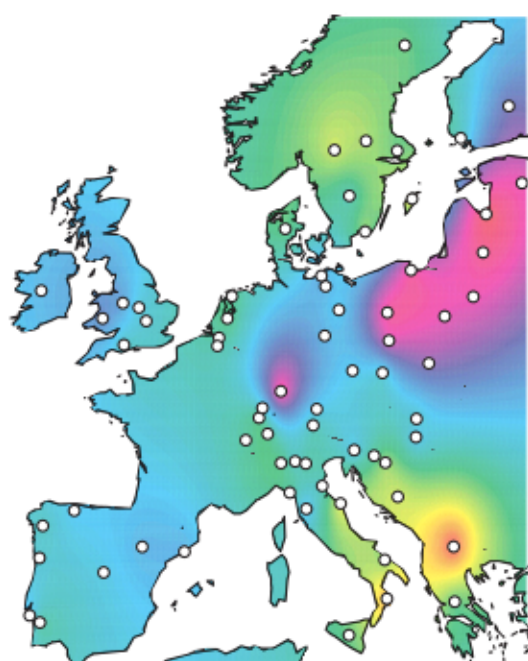

j

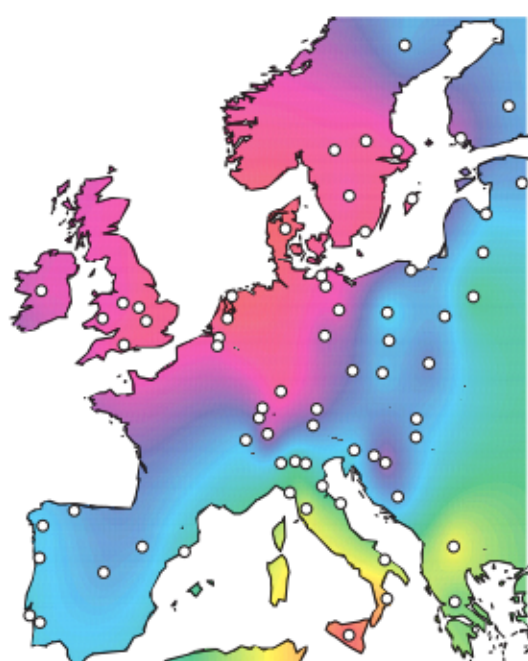

Supplement: Supplementary file 13 [file mmc13.pdf]
